# Supplementary material for: Foot and ankle Osteoarthritis and Cognitive impairment in retired UK Soccer players (FOCUS): protocol for a cross-sectional comparative study with general population controls
Source: BMJ Open. 2022 Apr 3;12(4):e054371. doi: 10.1136/bmjopen-2021-054371 (PMC8981329; doi:10.1136/bmjopen-2021-054371)

BMJ Open is committed to open peer review. As part of this commitment we make the peer review history of every article we publish publicly available.

When an article is published we post the peer reviewers' comments and the authors' responses online. We also post the versions of the paper that were used during peer review. These are the versions that the peer review comments apply to.

The versions of the paper that follow are the versions that were submitted during the peer review process. They are not the versions of record or the final published versions. They should not be cited or distributed as the published version of this manuscript.

BMJ Open is an open access journal and the full, final, typeset and author-corrected version of record of the manuscript is available on our site with no access controls, subscription charges or pay-per-view fees (http://bmjopen.bmj.com).

If you have any questions on BMJ Open's open peer review process please email info.bmjopen@bmj.com

BMJ Open

Foot and ankle Osteoarthritis and Cognitive impairment in retired UK Soccer players (FOCUS): protocol for a cross-sectional comparative study with general population controls

Journal:	*BMJ Open*
Manuscript ID	bmjopen-2021-054371
Article Type:	Protocol
Date Submitted by the Author:	11-Jun-2021

Complete List of Authors:	Espahbodi, Shima; University of Nottingham, Academic Rheumatology, School of Medicine; Centre for Sport, Exercise and Osteoarthritis Versus Arthritis, University of Nottingham<br>Fernandes, Gwen; University of Nottingham, Academic Rheumatology, School of Medicine; University of Bristol, Population Health Sciences<br>Hogervorst, Eef ; Loughborough University, NCSEM, School of Sport, Exercise and Health Sciences<br>Thanoon, Ahmed; University of Nottingham, Academic Rheumatology, School of Medicine; Centre for Sport, Exercise and Osteoarthritis Versus Arthritis, University of Nottingham<br>Batt, Mark; Centre for Sports, Exercise and Osteoarthritis Versus Arthritis, University of Nottingham; Nottingham University Hospitals NHS Trust, Sports Medicine<br>Fuller, Colin; Colin Fuller Consultancy Ltd<br>Fuller, Gordon; The University of Sheffield, Centre for Urgent and Emergency Research<br>Ferguson, Eamonn; University of Nottingham, Psychology<br>Bast, Tobias; University of Nottingham, Psychology; University of Nottingham, Neuroscience@Nottingham<br>Doherty, Michael ; University of Nottingham, Academic Rheumatology, School of Medicine; Centre for Sport, Exercise and Osteoarthritis Versus Arthritis, University of Nottingham<br>Zhang, Weiya; University of Nottingham, Academic Rheumatology, School of Medicine; Centre for Sport, Exercise and Osteoarthritis Versus Arthritis, University of Nottingham

Keywords:	EPIDEMIOLOGY, Dementia < NEUROLOGY, RHEUMATOLOGY

SCHOLARONE™
Manuscripts

Foot and ankle Osteoarthritis and Cognitive impairment in retired UK Soccer players (FOCUS): protocol for a cross-sectional comparative study with general population controls

Shima Espahbodi,[1,2,3] Gwen S Fernandes,[1,4] Eef Hogervorst,[5] Ahmed Thanoon,[1,2,3] Mark Batt,[2,10] Colin W Fuller,[6] Gordon Fuller,[7] Eamonn Ferguson,[3,8] Tobias Bast,[3,8,9] Michael Doherty,[1,2,3] Weiya Zhang[1,2,3,8,11]

[1] Academic Rheumatology, School of Medicine, University of Nottingham, UK

[2] Centre for Sport, Exercise and Osteoarthritis Research Versus Arthritis, University of Nottingham

[3] Pain Centre Versus Arthritis, University of Nottingham

[4] Population Health Sciences, Bristol Medical School, University of Bristol, UK

[5] NCSEM, School of Sport, Exercise and Health Sciences, Loughborough University

[6] Colin Fuller Consultancy Ltd, Sutton Bonington, UK

[7] Gordon Fuller, Centre for Urgent and Emergency Research, University of Sheffield

[8] School of Psychology, University of Nottingham

[9] Neuroscience@Nottingham, University of Nottingham

[10] Nottingham University Hospitals NHS Trust

[11] National Institution for Health Research (NIHR), Nottingham Biological Research Centre

Address for correspondence

shima.espahbodi1@nottingham.ac.uk

Word count: 4109

For peer review only

ABSTRACT

Introduction

Professional footballers commonly experience sports-related injury and repetitive microtrauma to the foot and ankle, placing them at risk of subsequent chronic pain and osteoarthritis (OA) of the foot and ankle. Similarly, repeated heading of the ball, head/neck injuries and concussion have been implicated in later development of neurodegenerative diseases such as dementia. A recent retrospective study found that death from neurodegenerative diseases was higher amongst former professional soccer players compared with age matched controls. However, well-designed lifetime studies are still needed to provide evidence regarding the prevalence of these conditions and their associated risk factors in retired professional football players compared to the general male population.

Objectives

To determine whether former professional male footballers have a higher prevalence than the general male population of: (1) foot/ankle pain and radiographic OA; and (2) cognitive and motor impairments associated with dementia and Parkinson's disease. Secondary objectives are to identify specific football-related risk factors such as head impact/concussion for neurodegenerative conditions and foot/ankle injuries for chronic foot/ankle pain and OA.

Methods and analysis

This is a cross-sectional, comparative study involving a questionnaire survey with sub-samples of responders being assessed for neurocognitive function by telephone assessment, and foot/ankle OA by radiographic examination. A sample of 900 adult, male, ex professional footballers will be recruited and compared with a control group of 1100 age-matched general population men between 40-100 years old. Prevalence will be estimated per group. Poisson regression will be performed to determine prevalence ratio between the populations and Logistic regression will be used to examine risk factors associated with each condition in footballers.

Ethics and dissemination

This study was approved by the East Midlands-Leicester Central Research Ethics Committee on 23 January 2020 (REC ref: 19/EM/0354). The study results will be disseminated at national and international meetings and submitted for peer-review publication.

Strengths and limitations of this study

- Largest study of head injuries/heading, concussion, ball type, and cognitive impairment in retired professional footballers versus controls in the UK.
- Largest study of foot/ankle injuries and OA/pain in retired professional footballers versus controls in the UK, with standardised foot/ankle radiographs obtained in a sample of each
- Case-control study design with control men recruited from a community-based population sample representative of men in the UK general population.

For peer review only

- Self-reporting of injuries and concussive symptomatology during careers/lifetime may be subject to recall bias, and non-involvement by individuals with severe cognitive impairment may cause left censorship.

For peer review only

INTRODUCTION

Professional footballers are at high-risk of sports-related injuries, including those to the head, thigh, knee and foot/ankle.[1] Over the past 25 years, Injuries to the thigh (23-26%), foot/ankle (20-23%) and knee (14-18%) have consistently been reported to be the three most common injury sites in professional football.[2-6] A study of one English Premier League club found that over a 4-season period, 20% of all injuries involved the foot/ankle with a mean return to play time of 54 days.[7] Injuries to the ankle lateral ligament complex, particularly the anterior talofibular ligament, were most frequent, accounting for 31% of all foot/ankle injuries. More minor repetitive impact-loading to the ankle/foot may also lead to joint and periarticular injury. However, despite the high incidence of acute injuries there is a paucity of robust evidence regarding subsequent osteoarthritis (OA) development and chronic pain in the foot/ankle joints of professional footballers. Some studies report a prevalence of ankle OA ranging from 12% - 17%.[1 8] However, these studies had small sample sizes without adequate control groups and were based on self-reported diagnosed ankle OA. Importantly, there are no published data on the prevalence of foot pain and foot OA in retired professional footballers. Consequently, foot and ankle OA is not formally recognised as an occupational disease for professional footballers, and there is a lack of evidence regarding the football-related risk factors associated with this condition.

Retired professional footballers with OA have a significantly lower health-related quality of life (QoL) compared to players without OA.[9] Ninety per cent of former professional footballers suffering with OA have moderate to severe joint pain, and 37% of these retired players reported moderate to severe depression and anxiety due to their medical condition.[1 10] In ex- UK footballers with OA, a higher frequency of disability and work-related disability has been reported compared to those without OA.[11] More recently we reported that depressive and anxiety symptoms in ex-footballers are comparable to that in the general population. However, ex-footballers reported significantly more sleep problems, negative mood profiles, more widespread bodily pain, and higher analgesic usage compared to controls.[12] Whilst OA might not be a life-threatening disease, its long-term effects on both the physical and mental health of a retired footballing population need further attention.

Professional footballers may also be at increased risk of developing neurodegenerative diseases such as dementias and Parkinson's disease, potentially arising from major neck/head injury, overt mild traumatic brain injury (mTBI) causing concussion, or repetitive microtrauma from heading the ball.[13-16] TBI has been identified as a risk factor for the development of dementia in the general population, specifically Alzheimer's, in a longitudinal cohort study (n=2.8 million) spanning 36 years.[17] In that study, moderate to severe TBI increased dementia risk across all ages, whereas mTBI (concussion) increased risk in those aged over 65 years.[17] Repetitive heading of the ball during matches and training sessions, head-to-head or head to elbow/knee/foot collisions between players, or accidental heading might additionally result in concussion, neuropsychiatric and cognitive deficits.[18] The recent FIELD study retrospectively compared mortality from neurodegenerative disease in 7676 former professional soccer

For peer review only

players with that amongst 23000 matched general population controls in Scotland. Former footballers had a 3.5 times higher death rate from neurodegenerative diseases compared to matched controls. Risk of death varied according to disease subtype and was 5 times higher in those with Alzheimer's and 2 times higher in those with Parkinson's respectively.[19] Whilst of considerable importance, these findings need to be confirmed in the life-time of professional footballers and matched controls. Still, little is known about the increased prevalence of neurodegenerative diseases and related risk factors among retired professional players. Recent literature continues to be conflicting and no study has provided conclusive evidence for the relationship between heading/head impacts and neurodegenerative diseases. Two recent systematic reviews support these conclusions and emphasise the need for high quality, large-scale prospective studies.[20 21]

Previously, we demonstrated a 2-3 times higher prevalence of knee OA in 1207 male ex-footballers compared to 4085 men in the general population[22] and identified knee injury and training load as the main football-specific factors for this.[23] We will use the same population samples for the Foot/ankle Osteoarthritis and Cognitive impairment in UK Soccer players (FOCUS) study. The primary objectives of this study are to determine whether, compared to age-matched men in the general population, ex-professional UK footballers have a higher prevalence of:

(1) foot/ankle pain and radiographic OA; and

(2) cognitive impairment, and neurodegenerative diseases particularly dementia and Parkinson's disease.

Secondary objectives are to identify factors associated with the risks of these conditions in footballers.

We have intentionally included investigations into foot/ankle OA together with neurodegenerative disease in this single cross-sectional study as it will allow both important research questions to be addressed concurrently. Furthermore, we anticipate that embedding questions and assessments on mental/cognitive function into a general health survey may increase the response rate and minimise the selection bias compared to asking questions in a survey focused solely on a single disease.

METHODS AND ANALYSIS

Study design

This will be a cross-sectional study comparing UK male ex-professional footballers and age-matched men in the general population. It will involve three discrete stages: (1) a postal questionnaire survey to all study participants; (2) a telephone assessment of cognitive function in a sub-sample of each group; and (3) a radiographic assessment of both feet and ankles in a sub-sample of each group. Figure 1 shows the study stages and procedures.

Participants and recruitment

For peer review only

The FOCUS footballer questionnaire, together with a Participant Information Sheet, Consent Form, stamped return envelope, and letter of endorsement from the Professional Footballers' Association (PFA) and Football Association (FA), will be posted across the UK to 900 ex-professional male footballers who previously participated in the Nottingham knee OA footballer study[22 24] and who indicated willingness to be contacted again for future studies. All are retired male footballers aged 40-100 years old, registered with the PFA or club player Association (League Club level). A slightly adapted questionnaire (e.g., no questions are included related to professional football), Participant Information Sheet, Consent Form, and stamped return envelope will be sent to 1100 age-matched men aged 40-100 in the East Midlands general population, who are on the Knee Pain in the Community (KPIC) database[25] and have indicated willingness to be contacted about new studies. This is the same source for general population controls as in our previous study on knee OA in footballers.[22] The Participant Information sheets state the entirely voluntary nature of the study with no inducements for participation. After four weeks a Reminder letter with questionnaire pack will be posted to those participants from whom there has been no response to the initial questionnaire pack.

Questionnaire responders who indicate willingness to be contacted further for telephone assessment and/or radiographs will be selected for stages 2 and 3, respectively. At least two hundred and fifty telephone assessments will be conducted first (randomly if possible), and the radiographs will be obtained later when COVID-19 restrictions permit us to utilize NHS Trust Radiology facilities. Exclusion criteria for the assessments include an inability to provide written informed consent (both assessments) and severe injury, amputation or neuropathic (Charcot) arthropathy of both feet (radiographic assessment only), and at the participant's request.

At least two hundred and fifty footballers will be invited to attend the radiology department of their nearest NHS collaborating hospital where foot/ankle radiographs will be undertaken. The selection will be based on first come first served, as well as the convenience to travel to the nearest hospitals. Currently we have five collaborating NHS hospitals including Nottingham, Salford, Leeds, Southampton, and Imperial College London. Similarly, at least 250 control men from a community-based population sample located within Nottingham and adjacent areas (Derbyshire, Lincolnshire, Leicestershire) representative of men in the general UK population, will have radiographs at Nottingham City Hospital campus. Return of completed questionnaires will be taken as consent to the questionnaire survey. For those participants agreeing to a telephone assessment consent will be taken verbally over the phone, after the participants have read and agreed to the consent form and patient information sheet posted to them. All participants attending for radiographs will be met by our research team who will obtain written informed consent prior to the taking of radiographs.

Questionnaire

For peer review only

Our FOCUS footballer questionnaire was designed to capture detailed information about foot/ankle pain, GP-diagnosed OA, significant injuries, surgery, and injections to the feet/ankles. Latter sections of the questionnaire capture detailed information about GP-diagnosed dementia, Alzheimer's and Parkinson's disease, the frequency of heading the ball during professional play, the type of ball, concussion history/symptoms, head injuries, and memory/cognition problems. For the general population male controls, the questionnaire includes all the same questions except for the absence of specific football-related questions. The layout/format of the questionnaire is consistent between footballers and control men.[25] Table 1 summarises the FOCUS questionnaire domains.

Table 1 FOCUS Questionnaire domains and measurements

Section Domains	Questions and Instruments
1. Demographic and current health	Date of birth, height, weight, list of comorbidities including diabetes, stroke, dementia, Alzheimer's, Parkinson's, depression, anxiety, HBP. Smoking and alcohol consumption. All current medication both prescribed and alternative/over the counter.
2. Bodily pain	Body pain mannequin for previous month's pain (current). fibromyalgia mannequin (WPI) and SSSs past week's pain.
3. Foot and ankle pain	Pain present in the feet and/or ankles in the past month (current) and/or past 3 months (chronic), age at pain onset, diagnosis of foot OA, region specific *significant* foot pain (ankle, mid-foot, big toe, other toes) and age range at onset of pain. Pain Detect Questionnaire and Pain Catastrophizing Scale.
4. Injuries and operations to feet/ankles	Significant football-related injury to ankles/feet/toes. Region specific (ankle, mid-foot, big toe, other toes) total number of significant football injuries throughout career, Type of Injury (e.g. ligament rupture) and age. Significant NON-football related injury ever (same questions as above).<br><br>Injections into ankles, type of injection (cortisone, anaesthetic, don't know, or other), greatest number of injections into each ankle in a season, and throughout career. Injections elsewhere in foot, where and how many. Surgical interventions to ankle or toes, type of procedure (e.g. fracture fixation), and age. If flat-footed as informed by health professional.
5. Hallux valgus	Current and constitutional toe alignment using line drawings (straight or bunion/degree of angulation).
6. How you feel	Hospital Anxiety and Depression Scale (HADS).
7. Quality of life	Short Form-36 (SF-36) Health Survey.
8. Heading during professional football	Heading frequency during a professional match and training session. General *heading play* (minor, moderate, major) during a match. Specific 'Only Heading' training sessions

For peer review only

	ever, frequency pre-season and during season. Type of football played with during career.
9. Concussion and head injuries	Concussion Definition. Concussion diagnosis from professional football play, number of episodes, symptoms. Concussion diagnosis from other/accident/fall, number, symptoms. Serious head injury, number, diagnosis. Frequency of Head impact NOT from heading not concussive. Concussion symptoms ever from professional play (unreported), frequency, symptoms. Concussion symptoms ever (unreported) outside of professional football e.g., fall, accident, number, symptoms.
10. Memory and thinking	Current Memory problems, worsening, causing difficulty with daily function, work or socially. The Test Your memory (TYM) Questionnaire.

HBP - High Blood Pressure; WPI - Widespread Pain Index; SSS - Symptom Severity Scale; OA - osteoarthritis Pain Detect Questionnaire, Pain Catastrophizing Scale, Hospital Anxiety and Depression Scale, SF-36 Health Survey and Test Your Memory Questionnaire are referenced in the main text.

The questionnaire requests information about the individual, their medical history, smoking and alcohol usage, and all current medications whether prescribed or over the counter. A body pain mannequin is used to record pain experienced for most days of the past month anywhere in the body, and the widespread pain index (WPI) and system severity scale (SSS)[26] are included to identify symptoms and likely diagnosis of fibromyalgia. Line drawings illustrating different regions of the foot (i.e. toes, mid-foot, and heel) allow participants to specify the site of any significant pain for most days in the past month and/or over a three month period. The Pain Detect Questionnaire (PDQ), modified to focus on ankle/foot pain only, will be used to screen for neuropathic type pain,[27] and the participants' thoughts and feelings during their foot pain experience will be measured with the Pain Catastrophizing Scale.[28]

The footballers are asked to report any football-related significant injury ever sustained to their feet, ankles, or toes as one which *caused pain for most days for at least a 3-month period* and the type of injury. A similar free text and tick-box table is included which asks for any significant non-football related injuries to the feet/ankles/toes. Information regarding injections into the ankles over the course of the players career are requested via a tick box table (type of injectable, location, side) and total number ever received in any season. A similar table with a free text and tick-box asks the footballers to report any surgery to their ankle and/or toes. Control participants are asked the same set of questions about significant injury, injections, and surgery to the ankles and/or toes but without mention of football. Current toe alignment and constitutional toe alignment (in their 20's) will be assessed in all participants using validated line drawings.[29]

Anxiety and depression symptoms will be assessed using the well validated Hospital and Anxiety Depression Scale (HADS).[30 31] Quality of Life (QoL) will be measured with the well-validated RAND 36-item Health Survey 1.0 (SF-36) adapted for the physical and mental health components respectively.[32]

For peer review only - http://bmjopen.bmj.com/site/about/guidelines.xhtml

Information related to heading the ball during footballers' professional careers will be captured with detailed questions developed with our retired footballer group (see Patient and Public Involvement). This will be followed with questions related to concussion diagnoses, symptomatology, and head injuries/impacts both during professional play and outside of professional football.[34] Control participants will be asked the same questions relating to head injury and concussions but without mention of football.

Final sections will capture information on memory/cognition and include the Test Your Memory (TYM) assessment,[35] which can be self-administered for use in postal surveys (personal communication JM Brown 2019). TYM is a short cognitive test for detecting Alzheimer's disease (AD) and other cognitive problems and is reported to be more sensitive for detecting mild AD than the Mini Mental State Examination (MMSE).[35]

Telephone cognitive assessments

Telephone assessments will follow a standardised script starting with the researchers introducing themselves and the study, checking that the participant is in a room without distractions, whether they use a hearing aid, confirming consent, their name and date of birth. An initial call will be made to book in the telephone assessment at a time convenient to each participant. The validated tools described below will be administered as per published procedures.

Originally developed as a dementia screen, the Telephone Interview for Cognitive Status-modified (TICS-m) is a test for cognitive function which can be administered over the telephone.[36 37] It includes four domains on (1) orientation; (2) registration (free recall), recent memory and delayed recall (memory); (3) attention/calculation; (4) comprehension, semantic memory and repetition (language). The 13-items (maximum score 39) include: (1) day, date, season, age, telephone number; (2) a 10-word list learning exercise then free recall of that word list; (3) subtractions and counting backwards; (4) responsive naming, current reigning monarch and prime minister; (5) word opposites; (6) repetition; and (7) delayed recall of 10-word list. TICS-m is well validated for detecting a range of mild to moderate cognitive disorders with comparable sensitivity and specificity as a screening tool for dementia and Alzheimer's.[38-40]

The Verbal Fluency Test (VFT) is a short task of verbal functioning and typically consists of a task called category (or semantic) fluency where participants are given one minute to name as many unique words as possible within a semantic category (animals in this study). The category 'animals' is often used, however alternatives are 'fruits and vegetables', 'cities and towns' and 'items of clothing'. The participant's score is the number of unique correct words produced in 60 seconds.[41] Word generation deficits in those with dementia, including Alzheimer's, have been well documented.[42]

The Hopkins Verbal Learning Test (HVLT)[43] is a quick and easy to administer tool which will be used to screen for dementia. It is a word-learning test measuring episodic verbal memory, consists of three trials of free-recall of a 12-item (word) list followed by yes/no recognition from the participant. After the

For peer review only

third learning trial, the participant is read 24 words and asked to say “yes” for each word that appeared on the recall list (12 targets) and “no” for each word that did not (12 distractors). The total immediate recall (reflecting learning ability, is obtained by repeating the same word list 3 times and adding up all correctly recalled words over the 3 trials) is calculated. Good validity and reliability have been demonstrated for the HVLT, and it is cross culturally applicable and well tolerated by elderly people.[44]

Along with cognitive measures, we have included the assessment of functional activities as impairment in these is a critical feature in the diagnosis of dementia.[45] Lawton’s Instrumental Activities Daily Living (IADL) scale is one of the most commonly used tools consisting of 8 items including 2 community items (mode of transport and shopping), 3 personal management (ability to use the telephone, handle money matters, and prepare one’s own medication) and 3 household items (doing housekeeping, laundry, and preparing food). Responses to each of the eight items in the scale are coded as 0 (unable), 1 (partially able), or 2 (able) and are the responses summed. Summary scores range from 0 (low function, dependent) to 8 (high function, independent).[46]

Radiographic assessment

Informed written consent will be obtained prior to radiographs which will be performed by each respective NHS Trust’s radiology staff. Radiographs will be taken bilaterally and weight-bearing for the foot and ankle separately. Foot dorsoplantar, medial oblique, and lateral views, and ankle anteroposterior views will be taken, as detailed in the Standard Operating Procedure (SOP). Examination and scoring of region-specific OA will be done of the hindfoot (ankle, subtalar), midfoot, and 1st metatarsophalangeal joint (MTPJ) using the La Trobe Foot Atlas and scorings.[47, 48] Those with a Kellgren and Lawrence score of less than 2 for any foot/ankle compartment and equivalent categories (i.e., completely normal, possible osteophyte or doubtful narrowing) will be defined as non-OA. Those who satisfy the definition of a KL score of greater than 2 (≥2) or equivalent for any compartment of either foot or ankle will be defined as OA.[49] Also, those with foot/ankle OA and concurrent foot/ankle pain will be defined as symptomatic foot/ankle OA.

Patient and public involvement

Questionnaire sections specific to professional football play were developed in partnership with ex-professional footballer groups, whilst those specific to sports-related concussion and memory/neurocognitive decline were developed in collaboration with concussion and dementia experts, (JM Brown).

Our retired player group contributed to the content, design and layout of the FOCUS Questionnaire. Through in-person meetings (SE), two retired professional players from Notts County Football Club directly advised (having had feedback from other retired players) specifically on the questions pertaining to:

- Heading frequency and bandings

For peer review only

- Training sessions, frequency and types
- Football types
- Types of injuries and question structure
- Head impacts and question content
- Questions on memory/cognitive function and the TYM test

The final questionnaire design was reviewed and trialled by 10 retired players at the PFA and 5 from Notts County Football Club.FC group. The time range for the ex-players to complete the questionnaire was 30-60 min and the feedback received was positive.

For peer review only

Outcome measures and statistical analysis

Continuous data will be presented as mean and standard deviations (SD) and categorical variables as frequencies and percentages. The statistical tests used to determine whether there is a significant difference between responses received from footballers and controls will be: t-test for normal distributions, Mann-Whitney U test for non-normal distributions, and Chi-square test for proportions with exact p values reported. Statistical significance will be set at $p<0.05$ with no allowances made for the number of statistical tests undertaken.

The prevalence of foot/ankle OA or neurodegenerative disease (and cognitive impairment) (self-reported or clinically assessed) with 95% confidence interval (CI) will be estimated per population. A Poisson regression model will be used to calculate prevalence risk ratio, adjusted for confounding factors such as age, body mass index (BMI), marital status, education, and social status. Linear (for continuous outcome) and logistic (for dichotomous outcome) regression analyses will be used to determine risk factors related to the outcome of interests within footballers. The results of this study will follow the STROBE guidelines for the reporting of observational studies[50].

Sample size calculation

We used a value of 14.3% prevalence for foot/ankle OA in the general population[51] and an odds ratio of 2 (to be clinically meaningful) between retired footballers and general population. The sample size was calculated using the z test and a multiple logistic regression model with a correlation factor R^2 of 0.3 among multiple risk factors/covariates using G*Power V.3.1.9.2.[52] Assuming footballers would have a greater risk of foot/ankle OA than controls (one tail), with power at 90% and significance at 0.05, 210 participants per group is required for foot/ankle OA outcome. We also calculated the sample size for neurodegenerative disease using the same method. According to 6.4% prevalence for neurodegenerative diseases in the general population[53], and 3.5 times more risk in the footballers reported recently in the FIELD study[19], the sample size required is 113 per group for this outcome with power at 90% and significance at 0.05. We will therefore use the larger sample size 210 per group for this study. We will recruit at least 250 retired professional footballers and 250 general population control men with a 16% leeway to account for potential dropouts and attrition.

For the postal questionnaire, we will contact all available retired professional footballers (n=900) and an age-matched sample of general population men (n=1100). Considering a 60% response rate based on our previous follow-up study[54], we would expect 540 footballers and 720 control men to respond to the questionnaire. This will provide sufficient samples from both groups to recruit the sub-sample of at least 250 participants per group required for the clinical assessments to achieve 90% power.

Discussion

To the best of our knowledge, this is the largest comparative study aiming to examine the prevalence and associated risk factors of the two common conditions, foot/ankle osteoarthritis (OA) and neurodegenerative disease in retired professional footballers versus age and sex matched general population controls. The study is expected to confirm the recent findings from the FIELD study concerning the increased risk of neurodegenerative disease in professional football players, but will also provide new evidence on whether concussion, head injury, ball type, number of games, training, position of play, are related to the increased risk of having this condition in later life. Unlike the FIELD study, this study will recruit the participants who are still alive to measure both severe disease phenotypes such as physician diagnosed dementia or Parkinson's as well as milder phenotypes such as mild cognitive impairment. Foot/ankle pain and OA will also be measured and compared between the two populations, which will further characterise the distribution of osteoarthritis and provide further evidence on the burden of foot/ankle OA in professional footballers over the general population controls following our previous study on knee OA[22]. An advantage of this 2 in 1 study is that it may help to mitigate the selection bias, which often occurs in a single disease study where people with the study condition may be more likely (or less likely) to participate. Comorbidities/multimorbidity such as cardiovascular diseases, diabetes, depression, chronic widespread pain, fibromyalgia and quality of life will also be collected to compare the general health status between footballers and matched controls.

However, the study has several limitations. Firstly, it is a cross-sectional study which can only confirm association but not causality. We have therefore planned to follow up the two cohorts for incidence of the diseases in the future. This has been considered for knee OA and multimorbidity following our previous study in knee OA, and we will be able to follow up the incidence of neurodegenerative disease/cognitive impairment and foot/ankle pain and OA as well in the future. Secondly, the study is not free from selection bias, e.g., footballers will not be selected randomly from all clubs but from our previous dataset. The same is true for the control population from the East Midlands because of the convenience of assessment in our local hospital. In addition, we are unable to randomly select participants who would like to have radiographs but need to take account of the distance to travel to the assigned NHS radiography departments. Thirdly, individuals who have developed marked cognitive and motor impairment may be unable or decline to respond to the questionnaire, leading to selection bias towards milder conditions. Last but not least, the COVID-19 pandemic has affected the project and protocols. We were unable to *conduct* face-to-face assessments of cognitive and motor function, or to

For peer review only

obtain radiographs (Nottingham, Southampton, Salford, Leeds, and Imperial College London). The project was temporarily halted following the national lockdown due to the COVID pandemic in March 2020. Following management committee meetings and advice from the University of Nottingham governance/Research Ethics Committee/Health Research Authority teams, it was decided to amend the original protocol from in-person assessments to telephone assessments in order to enable the study to begin. This approach was not possible for some of the neurocognitive assessments, and these were replaced with the TICS-m.[36 37] The TICS-m uses questions from MOCA and MMSE, with which it is highly correlated; it is a much shorter assessment and can be administered by telephone so is safer for all involved during the pandemic and lockdown. The VFT, HVLT, and IADL[41 43 45 46] were also retained as they can be administered by telephone, however the planned clinical neurological assessments (motor function) were removed.

Although the gold standard for testing cognitive function, and diagnosis of mild cognitive impairment (MCI) and dementia is face-to-face assessment using a battery of standardised and validated cognitive tests,[55] these are not without their drawbacks.[56] TICS-m is the most frequently used telephone-based cognitive screening test in medium-large studies and epidemiological surveys.[55] Key advantages include little evidence of ceiling and/or practice effects, and its better acceptance by participants who find the telephone interview less threatening than a face-to-face battery of cognitive tests in clinic.[36] Although hearing and communication may pose an issue for some, this can be mitigated by a skilled interviewer.[36 57] In contrast, the foot/ankle radiographs require NHS clinical radiology services, these have to be fully suspended until research activities within Hospital trusts are resumed.

In conclusion, we expect that the results of our study will establish prevalence of foot and ankle OA symptoms; radiographic prevalence of foot and ankle OA; and prevalence of cognitive impairment and neurodegenerative diseases in ex-professional footballers compared to general population controls. Thus, providing the much-needed answers following on from recent evidence that professional footballers had a 3.5 times higher death rate from neurodegenerative diseases compared with population controls.[19] Given that football is played professionally and recreationally in over 200 countries by more than 250 million people,[58] robust evidence on mental and physical health and well-being in retired professional footballers is a vital first step in establishing risk profiles, which will assist in developing effective prevention strategies for mitigating against longer-term health consequences.

Ethics and dissemination

All study aspects were approved by the East Midlands-Leicester Central Research Ethics Committee and HRA (REC ref: 19/EM/0354) on 23 January 2020. Study results will be submitted to the FA, Versus Arthritis, regulatory authorities, and peer reviewed journals for publication and presented at national and international conferences. Study participants will be provided with any resulting publications at their request.

Data statement

For peer review only

All data will be collected and stored in accordance with the Data Protection Act, 2018, University of Nottingham and NHS data handling and maintenance guidelines. The CRF will only collect the minimum required information for the purposes of the study. The study will be conducted in accordance with the ethical principles that have their origin in the Declaration of Helsinki, 1996; the principles of Good Clinical Practice and the UK Department of Health Policy Framework for Health and Social Care, 2017.

Authors' contributions

SE drafted, revised, finalised the manuscript, implemented (conducted) the study and NHS Trust collaborating centres. SE and GSF designed and developed the Footballer Questionnaires. GSF, MD, WZ conceptualised the study. All authors were involved in the design and development of the study protocols. SE, AT, EH and WZ collected data. All authors reviewed the draft and approved the final manuscript. WZ is the guarantor for the project.

Acknowledgments

We are grateful to Mr Les Bradd and all other retired professional footballers who gave their time and expertise to contribute to this study, including those from Notts County Football Club, The PFA, especially Mr Richard Jobson, and all our PPI group. We also extend our gratitude to Dr Jerry M Brown for his helpful discussion and invaluable advice on the TYM test, and our collaborators Professors Cathy Bowen, and Terry O'Neill, and Drs Richard Wakefield, Mo Aslam, and Lucy Gates. We are indebted to Dr Charlotte Cowie for her guidance and support throughout the past few years. We wish to acknowledge the FA, the PFA, Versus Arthritis, and the University of Nottingham for supporting this study, and King Abdulaziz University, Jeddah, Saudi Arabia for sponsoring AT's PhD programme. Finally, we are thankful to our participants both from the retired professional football players and those from the general population dwelling adults from the East-Midlands. Without their pertinent and persistent support over the past years, we would not be able to undertake this study especially during the COVID-19 pandemic/lockdown.

Figure Legend

Figure 1: Flow chart of FOCUS study stages and procedures. *Minimum sample size according to sample size calculation.

Funding statement

This work is supported by grants from Versus Arthritis, grant number (21595) and The Football Association (FA), grant number (not applicable).

Competing interests.

CF provides consultancy services to the English Premier League.

For peer review only

Patient consent for publication. Not required

Provenance and peer review. Not commissioned, externally peer reviewed for ethical and funding approval prior to submission.

References

1. Drawer S, Fuller CW. Propensity for osteoarthritis and lower limb joint pain in retired professional soccer players. *Br J Sports Med* 2001;35(6):402-8. doi: 10.1136/bjsm.35.6.402 [published Online First: 2001/12/01]
2. Hawkins RD, Fuller CW. A prospective epidemiological study of injuries in four English professional football clubs. *British journal of sports medicine* 1999;33(3):196-203. doi: 10.1136/bjsm.33.3.196
3. Ekstrand J, Hägglund M, Waldén M. Injury incidence and injury patterns in professional football: the UEFA injury study. *Br J Sports Med* 2011;45(7):553-8. doi: 10.1136/bjsm.2009.060582 [published Online First: 2009/06/26]
4. López-Valenciano A, Ruiz-Pérez I, Garcia-Gómez A, et al. Epidemiology of injuries in professional football: a systematic review and meta-analysis. *British journal of sports medicine* 2020;54(12):711. doi: 10.1136/bjsports-2018-099577
5. Chomiak J, Junge A, Peterson L, et al. Severe injuries in football players. Influencing factors. *The American journal of sports medicine* 2000;28(5 Suppl):S58-68. doi: 10.1177/28.suppl_5.s-58 [published Online First: 2000/10/14]
6. Waldén M, Hägglund M, Ekstrand J. Time-trends and circumstances surrounding ankle injuries in men's professional football: an 11-year follow-up of the UEFA Champions League injury study. *Br J Sports Med* 2013;47(12):748-53. doi: 10.1136/bjsports-2013-092223 [published Online First: 2013/07/03]
7. Jain N, Murray D, Kemp S, et al. Frequency and trends in foot and ankle injuries within an English Premier League Football Club using a new impact factor of injury to identify a focus for injury prevention. *Foot Ankle Surg* 2014;20(4):237-40. doi: 10.1016/j.fas.2014.05.004 [published Online First: 2014/12/03]
8. Kuijt MT, Inklaar H, Gouttebarge V, et al. Knee and ankle osteoarthritis in former elite soccer players: a systematic review of the recent literature. *J Sci Med Sport* 2012;15(6):480-7. doi: 10.1016/j.jsams.2012.02.008 [published Online First: 2012/05/11]
9. Gouttebarge V, Aoki H, Kerkhoffs G. Lower extremity osteoarthritis is associated with lower health-related quality of life among retired professional footballers. *Phys Sportsmed* 2018;46(4):471-76. doi: 10.1080/00913847.2018.1451718 [published Online First: 2018/03/13]
10. Turner A, Barlow J, Ilbery B. Play Hurt, Live Hurt: Living with and Managing Osteoarthritis from the Perspective of Ex-professional Footballers. *J Health Psychol* 2002;7(3):285-301. doi: 10.1177/1359105302007003222 [published Online First: 2002/05/01]
11. Turner AP, Barlow JH, Heathcote-Elliott C. Long term health impact of playing professional football in the United Kingdom. *British journal of sports medicine* 2000;34(5):332-36. doi: 10.1136/bjsm.34.5.332
12. Fernandes GS, Parekh SM, Moses J, et al. Depressive symptoms and the general health of retired professional footballers compared with the general population in the UK: a case-control study. *BMJ Open* 2019;9(9):e030056. doi: 10.1136/bmjopen-2019-030056 [published Online First: 2019/09/11]
13. Rutherford A, Stewart W, Bruno D. Heading for trouble: is dementia a game changer for football? *British journal of sports medicine* 2019;53(6):321-22. doi: 10.1136/bjsports-2017-097627
14. Guskiewicz KM, Marshall SW, Bailes J, et al. Association between recurrent concussion and late-life cognitive impairment in retired professional football players. *Neurosurgery* 2005;57(4):719-26; discussion 19-26. doi: 10.1093/neurosurgery/57.4.719 [published Online First: 2005/10/22]
15. Lee MJ. Increase risk for neurodegenerative diseases in professional athletes. University of Boston, 2015.
16. Ling H, Morris HR, Neal JW, et al. Mixed pathologies including chronic traumatic encephalopathy account for dementia in retired association football (soccer) players. *Acta Neuropathol*

For peer review only

2017;133(3):337-52. doi: 10.1007/s00401-017-1680-3 [published Online First: 2017/02/17]

17. Fann JR, Ribe AR, Pedersen HS, et al. Long-term risk of dementia among people with traumatic brain injury in Denmark: a population-based observational cohort study. *Lancet Psychiatry* 2018;5(5):424-31. doi: 10.1016/s2215-0366(18)30065-8 [published Online First: 2018/04/15]
18. Fuller CW, Junge A, Dvorak J. A six year prospective study of the incidence and causes of head and neck injuries in international football. *Br J Sports Med* 2005;39 Suppl 1(Suppl 1):i3-9. doi: 10.1136/bjsm.2005.018937 [published Online First: 2005/07/28]
19. Mackay DF, Russell ER, Stewart K, et al. Neurodegenerative Disease Mortality among Former Professional Soccer Players. *New England Journal of Medicine* 2019;381(19):1801-08. doi: 10.1056/NEJMoa1908483
20. Kontos AP, Braithwaite R, Chrisman SPD, et al. Systematic review and meta-analysis of the effects of football heading. *Br J Sports Med* 2017;51(15):1118-24. doi: 10.1136/bjsports-2016-096276 [published Online First: 2016/12/23]
21. Tarnutzer AA, Straumann D, Brugger P, et al. Persistent effects of playing football and associated (subconcussive) head trauma on brain structure and function: a systematic review of the literature. *Br J Sports Med* 2017;51(22):1592-604. doi: 10.1136/bjsports-2016-096593 [published Online First: 2016/11/07]
22. Fernandes GS, Parekh SM, Moses J, et al. Prevalence of knee pain, radiographic osteoarthritis and arthroplasty in retired professional footballers compared with men in the general population: a cross-sectional study. *Br J Sports Med* 2018;52(10):678-83. doi: 10.1136/bjsports-2017-097503 [published Online First: 2017/11/05]
23. Parekh SM, Fernandes GS, Moses JP, et al. Risk Factors for Knee Osteoarthritis in Retired Professional Footballers: A Cross-Sectional Study. *Clinical journal of sport medicine : official journal of the Canadian Academy of Sport Medicine* 2019 doi: 10.1097/JSM.0000000000000742 [published Online First: 2019/06/04]
24. Faul F, Erdfelder E, Lang A-G, et al. G*Power 3: A flexible statistical power analysis program for the social, behavioral, and biomedical sciences. *Behavior Research Methods* 2007;39(2):175-91. doi: 10.3758/BF03193146
25. Fernandes GS, Sarmanova A, Warner S, et al. Knee pain and related health in the community study (KPIC): a cohort study protocol. *BMC Musculoskelet Disord* 2017;18(1):404. doi: 10.1186/s12891-017-1761-4
26. Wolfe F, Clauw DJ, Fitzcharles MA, et al. Fibromyalgia criteria and severity scales for clinical and epidemiological studies: a modification of the ACR Preliminary Diagnostic Criteria for Fibromyalgia. *J Rheumatol* 2011;38(6):1113-22. doi: 10.3899/jrheum.100594 [published Online First: 2011/02/03]
27. Freynhagen R, Baron R, Gockel U, et al. painDETECT: a new screening questionnaire to identify neuropathic components in patients with back pain. *Curr Med Res Opin* 2006;22(10):1911-20. doi: 10.1185/030079906x132488 [published Online First: 2006/10/07]
28. Sullivan MJL, Bishop SR, Pivik J. The Pain Catastrophizing Scale: Development and validation. *Psychological Assessment* 1995;7(4):524-32. doi: 10.1037/1040-3590.7.4.524
29. Chatterton BD, Muller S, Thomas MJ, et al. Inter and intra-rater repeatability of the scoring of foot pain drawings. *Journal of Foot and Ankle Research* 2013;6(1):44. doi: 10.1186/1757-1146-6-44
30. Spinhoven P, Ormel J, Sloekers PP, et al. A validation study of the Hospital Anxiety and Depression Scale (HADS) in different groups of Dutch subjects. *Psychol Med* 1997;27(2):363-70. doi: 10.1017/s0033291796004382 [published Online First: 1997/03/01]
31. Bjelland I, Dahl AA, Haug TT, et al. The validity of the Hospital Anxiety and Depression Scale. An updated literature review. *J Psychosom Res* 2002;52(2):69-77. doi: 10.1016/s0022-3999(01)00296-3 [published Online First: 2002/02/08]
32. Ware JE, Jr., Sherbourne CD. The MOS 36-item short-form health survey (SF-36). I. Conceptual framework and item selection. *Med Care* 1992;30(6):473-83. [published Online First: 1992/06/11]
33. Medical Outcomes Study: 36-item Short Form Survey [Available from: https://www.rand.org/health-care/surveys_tools/mos/36-item-short-form.html.
34. McCrory P, Meeuwisse W, Dvorak J, et al. Consensus statement on concussion in sport—the 5th international conference on concussion in sport held in Berlin, October 2016. *British journal of sports medicine* 2017;51(11):838-47. doi: 10.1136/bjsports-2017-097699

For peer review only

35. Brown J, Pengas G, Dawson K, et al. Self administered cognitive screening test (TYM) for detection of Alzheimer's disease: cross sectional study. *Bmj* 2009;338:b2030. doi: 10.1136/bmj.b2030 [published Online First: 2009/06/11]
36. Brandt J, Spencer M, Folstein M. The Telephone Interview for Cognitive Status. *Neuropsychiatry, Neuropsychology, & Behavioral Neurology* 1988;1(2):111-17.
37. de Jager CA, Budge MM, Clarke R. Utility of TICS-M for the assessment of cognitive function in older adults. *Int J Geriatr Psychiatry* 2003;18(4):318-24. doi: 10.1002/gps.830 [published Online First: 2003/04/04]
38. Smith MM, Tremont G, Ott BR. A review of telephone-administered screening tests for dementia diagnosis. *Am J Alzheimers Dis Other Demen* 2009;24(1):58-69. doi: 10.1177/1533317508327586 [published Online First: 2009/01/01]
39. Welsh KA, Breitner JC, Magruder-Habib KM. Detection of dementia in the elderly using telephone screening of cognitive status. *Neuropsychiatry, Neuropsychology, & Behavioral Neurology* 1993;6(2):103-10.
40. Wolfson C, Kirkland SA, Raina PS, et al. Telephone-administered cognitive tests as tools for the identification of eligible study participants for population-based research in aging. *Can J Aging* 2009;28(3):251-9. doi: 10.1017/s0714980809990092 [published Online First: 2009/10/29]
41. Alegret M, Peretó M, Pérez A, et al. The Role of Verb Fluency in the Detection of Early Cognitive Impairment in Alzheimer's Disease. *J Alzheimers Dis* 2018;62(2):611-19. doi: 10.3233/jad-170826 [published Online First: 2018/02/27]
42. Gomez RG, White DA. Using verbal fluency to detect very mild dementia of the Alzheimer type. *Arch Clin Neuropsychol* 2006;21(8):771-5. doi: 10.1016/j.acn.2006.06.012 [published Online First: 2006/10/03]
43. Brandt J. The hopkins verbal learning test: Development of a new memory test with six equivalent forms. *Clinical Neuropsychologist* 1991;5(2):125-42. doi: 10.1080/13854049108403297
44. Hogervorst E, Combrinck M, Lapuerta P, et al. The Hopkins Verbal Learning Test and screening for dementia. *Dement Geriatr Cogn Disord* 2002;13(1):13-20. doi: 10.1159/000048628 [published Online First: 2001/12/04]
45. Association AP. Diagnostic and Statistical Manual of Metal Disorders. Fifth Edition ed. Arlington VA: American Psychiatric Publishing 2013.
46. Lawton MP, Brody EM. Assessment of Older People: Self-Maintaining and Instrumental Activities of Daily Living1. *The Gerontologist* 1969;9(3_Part_1):179-86. doi: 10.1093/geront/9.3_Part_1.179
47. Menz HB, Munteanu SE, Landorf KB, et al. Radiographic classification of osteoarthritis in commonly affected joints of the foot. *Osteoarthritis Cartilage* 2007;15(11):1333-8. doi: 10.1016/j.joca.2007.05.007 [published Online First: 2007/07/13]
48. Kraus VB, Kilfoil TM, Hash TW, 2nd, et al. Atlas of radiographic features of osteoarthritis of the ankle and hindfoot. *Osteoarthritis Cartilage* 2015;23(12):2059-85. doi: 10.1016/j.joca.2015.08.008 [published Online First: 2015/09/01]
49. Kellgren JH, Lawrence JS. Radiological assessment of osteo-arthrosis. *Ann Rheum Dis* 1957;16(4):494-502. doi: 10.1136/ard.16.4.494 [published Online First: 1957/12/01]
50. von Elm E, Altman DG, Egger M, et al. The Strengthening the Reporting of Observational Studies in Epidemiology (STROBE) statement: guidelines for reporting observational studies. *The Lancet* 2007;370(9596):1453-57. doi: 10.1016/S0140-6736(07)61602-X
51. Roddy E, Thomas MJ, Marshall M, et al. The population prevalence of symptomatic radiographic foot osteoarthritis in community-dwelling older adults: cross-sectional findings from the Clinical Assessment Study of the Foot. *Annals of the Rheumatic Diseases* 2015;74(1):156. doi: 10.1136/annrheumdis-2013-203804
52. Faul F, Erdfelder E, Buchner A, et al. Statistical power analyses using G*Power 3.1: Tests for correlation and regression analyses. *Behavior Research Methods* 2009;41(4):1149-60. doi: 10.3758/BRM.41.4.1149
53. Lobo A, Launer LJ, Fratiglioni L, et al. Prevalence of dementia and major subtypes in Europe: A collaborative study of population-based cohorts. Neurologic Diseases in the Elderly Research Group. *Neurology* 2000;54(11 Suppl 5):S4-9. [published Online First: 2000/06/15]
54. Ingham SL, Zhang W, Doherty SA, et al. Incident knee pain in the Nottingham community: a 12-year retrospective cohort study. *Osteoarthritis Cartilage* 2011;19(7):847-52. doi: 10.1016/j.joca.2011.03.012
55. Herr M, Ankri J. A critical review of the use of telephone tests to identify cognitive impairment in epidemiology and clinical research. *J Telemed Telecare* 2013;19(1):45-54. doi: 10.1177/1357633x12474962 [published Online First: 2013/02/08]

For peer review only - http://bmjopen.bmj.com/site/about/guidelines.xhtml

56. Pachana NA, Alpass FM, Blakey JA, et al. A comparison of the MMSE and the TICS-m in hearing-impaired older adults. *Australasian Journal on Ageing* 2006;25(2):89-93. doi: https://doi.org/10.1111/j.1741-6612.2006.00156.x
57. Kliegel M, Martin M, Jäger T. Development and validation of the Cognitive Telephone Screening Instrument (COGTEL) for the assessment of cognitive function across adulthood. *J Psychol* 2007;141(2):147-70. doi: 10.3200/jrlp.141.2.147-172 [published Online First: 2007/05/08]
58. Kunz M. 265 million playing football. FIFA Magazine. Zurich: FIFA 2007 [Available from: https://condorperformance.com/wp-content/uploads/2020/02/emaga_9384_10704.pdf.

For peer review only

Figure 1 Flow chart of FOCUS study stages and procedures. * Minimum sample size according to the sample size calculation

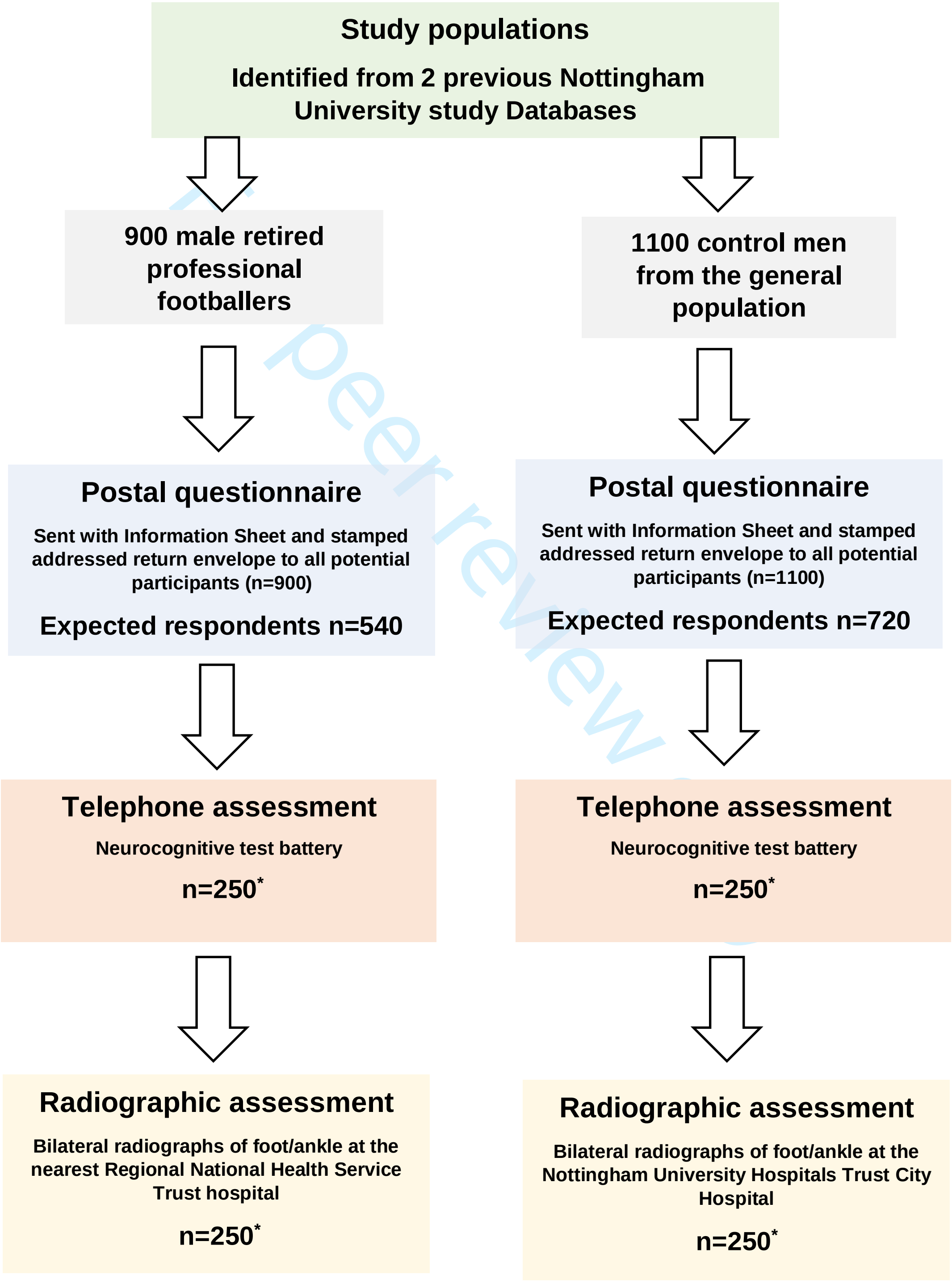

BMJ Open

Foot and ankle Osteoarthritis and Cognitive impairment in retired UK Soccer players (FOCUS): protocol for a cross-sectional comparative study with general population controls

Journal:	*BMJ Open*
Manuscript ID	bmjopen-2021-054371.R1
Article Type:	Protocol
Date Submitted by the Author:	19-Dec-2021

Complete List of Authors:	Espahbodi, Shima; University of Nottingham, Academic Rheumatology, School of Medicine; Centre for Sport, Exercise and Osteoarthritis Versus Arthritis, University of Nottingham<br>Fernandes, Gwen; University of Nottingham, Academic Rheumatology, School of Medicine; University of Bristol, Population Health Sciences<br>Hogervorst, Eef ; Loughborough University, NCSEM, School of Sport, Exercise and Health Sciences<br>Thanoon, Ahmed; University of Nottingham, Academic Rheumatology, School of Medicine; Centre for Sport, Exercise and Osteoarthritis Versus Arthritis, University of Nottingham<br>Batt, Mark; Centre for Sports, Exercise and Osteoarthritis Versus Arthritis, University of Nottingham; Nottingham University Hospitals NHS Trust, Sports Medicine<br>Fuller, Colin; Colin Fuller Consultancy Ltd<br>Fuller, Gordon; The University of Sheffield, Centre for Urgent and Emergency Research<br>Ferguson, Eamonn; University of Nottingham, Psychology<br>Bast, Tobias; University of Nottingham, Psychology; University of Nottingham, Neuroscience@Nottingham<br>Doherty, Michael ; University of Nottingham, Academic Rheumatology, School of Medicine; Centre for Sport, Exercise and Osteoarthritis Versus Arthritis, University of Nottingham<br>Zhang, Weiya; University of Nottingham, Academic Rheumatology, School of Medicine; Centre for Sport, Exercise and Osteoarthritis Versus Arthritis, University of Nottingham

**Primary Subject Heading**:	Sports and exercise medicine
Secondary Subject Heading:	Epidemiology
Keywords:	EPIDEMIOLOGY, Dementia < NEUROLOGY, RHEUMATOLOGY, SPORTS MEDICINE

For peer review only - http://bmjopen.bmj.com/site/about/guidelines.xhtml

Foot and ankle Osteoarthritis and Cognitive impairment in retired UK Soccer players (FOCUS): protocol for a cross-sectional comparative study with general population controls

Shima Espahbodi,[1,2,3] Gwen S Fernandes,[1,4] Eef Hogervorst,[5] Ahmed Thanoon,[1,2,3.] Mark Batt,[2,10] Colin W Fuller,[6] Gordon Fuller,[7] Eamonn Ferguson,[3,8] Tobias Bast,[3,8,9] Michael Doherty,[1,2,3] Weiya Zhang[1,2,3,8,11]

[1] Academic Rheumatology, School of Medicine, University of Nottingham, UK

[2] Centre for Sport, Exercise and Osteoarthritis Research Versus Arthritis, University of Nottingham

[3] Pain Centre Versus Arthritis, University of Nottingham

[4] Population Health Sciences, Bristol Medical School, University of Bristol, UK

[5] NCSEM, School of Sport, Exercise and Health Sciences, Loughborough University

[6] Colin Fuller Consultancy Ltd, Sutton Bonington, UK

[7] Gordon Fuller, Centre for Urgent and Emergency Research, University of Sheffield

[8] School of Psychology, University of Nottingham

[9] Neuroscience@Nottingham, University of Nottingham

[10] Nottingham University Hospitals NHS Trust

[11] National Institution for Health Research (NIHR), Nottingham Biological Research Centre

Address for correspondence

shima.espahbodi1@nottingham.ac.uk

Word count: 4109

For peer review only

ABSTRACT

Introduction

Professional footballers commonly experience sports-related injury and repetitive microtrauma to the foot and ankle, placing them at risk of subsequent chronic pain and osteoarthritis (OA) of the foot and ankle. Similarly, repeated heading of the ball, head/neck injuries and concussion have been implicated in later development of neurodegenerative diseases such as dementia. A recent retrospective study found that death from neurodegenerative diseases was higher amongst former professional soccer players compared with age matched controls. However, well-designed lifetime studies are still needed to provide evidence regarding the prevalence of these conditions and their associated risk factors in retired professional football players compared to the general male population.

Objectives

To determine whether former professional male footballers have a higher prevalence than the general male population of: (1) foot/ankle pain and radiographic OA; and (2) cognitive and motor impairments associated with dementia and Parkinson's disease. Secondary objectives are to identify specific football-related risk factors such as head impact/concussion for neurodegenerative conditions and foot/ankle injuries for chronic foot/ankle pain and OA.

Methods and analysis

This is a cross-sectional, comparative study involving a questionnaire survey with sub-samples of responders being assessed for neurocognitive function by telephone assessment, and foot/ankle OA by radiographic examination. A sample of 900 adult, male, ex professional footballers will be recruited and compared with a control group of 1100 age-matched general population men between 40-100 years old. Prevalence will be estimated per group. Poisson regression will be performed to determine prevalence ratio between the populations and Logistic regression will be used to examine risk factors associated with each condition in footballers.

Ethics and dissemination

This study was approved by the East Midlands-Leicester Central Research Ethics Committee on 23 January 2020 (REC ref: 19/EM/0354). The study results will be disseminated at national and international meetings and submitted for peer-review publication.

Strengths and limitations of this study

- Largest study of head injuries/heading, concussion, ball type, and cognitive impairment in retired professional footballers versus controls in the UK.
- Largest study of foot/ankle injuries and OA/pain in retired professional footballers versus controls in the UK, with standardised foot/ankle radiographs obtained in a sample of each
- Case-control study design with control men recruited from a community-based population sample representative of men in the UK general population.

For peer review only

- Self-reporting of injuries and concussive symptomatology during careers/lifetime may be subject to recall bias, and non-involvement by individuals with severe cognitive impairment may cause left censorship.

For peer review only

INTRODUCTION

Professional footballers are at high-risk of sports-related injuries, including those to the head, thigh, knee and foot/ankle.[1] Over the past 25 years, the prevalence of injuries to the thigh (23-26%), foot/ankle (20-23%) and knee (14-18%) have consistently been reported to be the three most common injury sites in professional football.[2-6] A study of one English Premier League club found that over a 4-season period, 20% of all injuries involved the foot/ankle with a mean return to play time of 54 days.[7] Injuries to the ankle lateral ligament complex, particularly the anterior talofibular ligament, were most frequent, accounting for 31% of all foot/ankle injuries. More minor repetitive impact-loading to the ankle/foot may also lead to joint and periarticular injury. However, despite the high incidence of acute injuries there is a paucity of robust evidence regarding subsequent osteoarthritis (OA) development and chronic pain in the foot/ankle joints of professional footballers. Some studies report a prevalence of ankle OA ranging from 12% - 17%.[1 8] However, these studies had small sample sizes without adequate control groups and were based on self-reported diagnosed ankle OA. Importantly, there are no published data on the prevalence of foot pain and foot OA in retired professional footballers. Consequently, foot and ankle OA is not formally recognised as an occupational disease for professional footballers, and there is a lack of evidence regarding the football-related risk factors associated with this condition.

Retired professional footballers with OA have a significantly lower health-related quality of life (QoL) compared to players without OA.[9] Ninety per cent of former professional footballers suffering with OA have moderate to severe joint pain, and 37% of these retired players reported moderate to severe depression and anxiety due to their medical condition.[1 10] In ex- UK footballers with OA, a higher frequency of disability and work-related disability has been reported compared to those without OA.[11] More recently we reported that depressive and anxiety symptoms in ex-footballers are comparable to that in the general population. However, ex-footballers reported significantly more sleep problems, negative mood profiles, more widespread bodily pain, and higher analgesic usage compared to controls.[12] Whilst OA might not be a life-threatening disease, its long-term effects on both the physical and mental health of a retired footballing population need further attention.

Professional footballers may also be at increased risk of developing neurodegenerative diseases such as dementias and Parkinson's disease, potentially arising from major neck/head injury, overt mild traumatic brain injury (mTBI) causing concussion, or repetitive microtrauma from heading the ball.[13-16] TBI has been identified as a risk factor for the development of dementia in the general population, specifically Alzheimer's, in a longitudinal cohort study (n=2.8 million) spanning 36 years.[17] In that study, moderate to severe TBI increased dementia risk across all ages, whereas mTBI (concussion) increased risk in those aged over 65 years.[17] Repetitive heading of the ball during matches and training sessions, head-to-head or head to elbow/knee/foot collisions between players, or accidental heading might additionally result in concussion, neuropsychiatric and cognitive deficits.[18] The recent FIELD study retrospectively compared mortality from neurodegenerative disease in 7676 former professional soccer

For peer review only

players with that amongst 23000 matched general population controls in Scotland. Former footballers had a 3.5 times higher death rate from neurodegenerative diseases compared to matched controls. Risk of death varied according to disease subtype and was 5 times higher in those with Alzheimer's and 2 times higher in those with Parkinson's respectively.[19] Whilst of considerable importance, these findings need to be confirmed in the life-time of professional footballers and matched controls. Still, little is known about the increased prevalence of neurodegenerative diseases and related risk factors among retired professional players. Recent literature continues to be conflicting and no study has provided conclusive evidence for the relationship between heading/head impacts and neurodegenerative diseases. Two recent systematic reviews support these conclusions and emphasise the need for high quality, large-scale prospective studies.[20 21]

Previously, we demonstrated a 2-3 times higher prevalence of knee OA in 1207 male ex-footballers compared to 4085 men in the general population[22] and identified knee injury and training load as the main football-specific factors for this.[23] We will use the same population samples for the Foot/ankle Osteoarthritis and Cognitive impairment in UK Soccer players (FOCUS) study. The primary objectives of this study are to determine whether, compared to age-matched men in the general population, ex-professional UK footballers have a higher prevalence of:

(1) foot/ankle pain and radiographic OA; and

(2) cognitive impairment, and neurodegenerative diseases particularly dementia and Parkinson's disease.

Secondary objectives are to identify factors associated with the risks of these conditions in footballers.

We have intentionally included investigations into foot/ankle OA together with neurodegenerative disease in this single cross-sectional study as it will allow both important research questions to be addressed concurrently. Furthermore, we anticipate that embedding questions and assessments on mental/cognitive function into a general health survey may increase the response rate and minimise the selection bias compared to asking questions in a survey focused solely on a single disease.

METHODS AND ANALYSIS

Study design

This will be a cross-sectional study comparing UK male ex-professional footballers and age-matched men in the general population. It will involve three discrete stages: (1) a postal questionnaire survey to all study participants; (2) a telephone assessment of cognitive function in a sub-sample of each group; and (3) a radiographic assessment of both feet and ankles in a sub-sample of each group. The planned start and end dates for the study are August 2020-December 2022. Figure 1 shows the study stages and procedures.

For peer review only

Participants and recruitment

The FOCUS footballer questionnaire, together with a Participant Information Sheet, Consent Form, stamped return envelope, and letter of endorsement from the Professional Footballers' Association (PFA) and Football Association (FA), will be posted across the UK to 900 ex-professional male footballers who previously participated in the Nottingham knee OA footballer study[22 24] and who indicated willingness to be contacted again for future studies. All are retired male footballers aged 40-100 years old, registered with the PFA or club player Association (League Club level). A slightly adapted questionnaire (e.g., no questions are included related to professional football), Participant Information Sheet, Consent Form, and stamped return envelope will be sent to 1100 age-matched men aged 40-100 in the East Midlands general population, who are on the Knee Pain in the Community (KPIC) database[25] and have indicated willingness to be contacted about new studies. This is the same source for general population controls as in our previous study on knee OA in footballers.[22] The Participant Information sheets state the entirely voluntary nature of the study with no inducements for participation. After four weeks a Reminder letter with questionnaire pack will be posted to those participants from whom there has been no response to the initial questionnaire pack.

Questionnaire responders who indicate willingness to be contacted further for telephone assessment and/or radiographs will be selected for stages 2 and 3, respectively. At least two hundred and fifty telephone assessments will be conducted first, and the radiographs will be obtained later when COVID-19 restrictions permit us to utilize NHS Trust Radiology facilities. Exclusion criteria for the assessments include an inability to provide written informed consent (both assessments) and severe injury, amputation or neuropathic (Charcot) arthropathy of both feet (radiographic assessment only), and at the participant's request.

At least two hundred and fifty footballers will be invited to attend the radiology department of their nearest NHS collaborating hospital where foot/ankle radiographs will be undertaken. The selection will be based on first come first served, as well as the convenience to travel to the nearest hospitals. Currently we have five collaborating NHS hospitals including Nottingham, Salford, Leeds, Southampton, and Imperial College London. Similarly, at least 250 control men from a community-based population sample located within Nottingham and adjacent areas (Derbyshire, Lincolnshire, Leicestershire) representative of men in the general UK population, will have radiographs at Nottingham City Hospital campus. Return of completed questionnaires will be taken as consent to the questionnaire survey. For those participants agreeing to a telephone assessment consent will be taken verbally over the phone, after the participants have read and agreed to the consent form and patient information sheet posted to them. All participants attending for radiographs will be met by our research team who will obtain written informed consent prior to the taking of radiographs.

Questionnaire

For peer review only

Our FOCUS footballer questionnaire was designed to capture detailed information about foot/ankle pain, GP-diagnosed OA, significant injuries, surgery, and injections to the feet/ankles. Latter sections of the questionnaire capture detailed information about GP-diagnosed dementia, Alzheimer's and Parkinson's disease, the frequency of heading the ball during professional play, the type of ball, concussion history/symptoms, head injuries, and memory/cognition problems. For the general population male controls, the questionnaire includes all the same questions except for the absence of specific football-related questions. The layout/format of the questionnaire is consistent between footballers and control men.[25] Table 1 summarises the FOCUS questionnaire domains.

Table 1 FOCUS Questionnaire domains and measurements

Section Domains	Questions and Instruments
1. Demographic and current health	Date of birth, height, weight, list of comorbidities including diabetes, stroke, dementia, Alzheimer's, Parkinson's, depression, anxiety, HBP. Smoking and alcohol consumption. All current medication both prescribed and alternative/over the counter.
2. Bodily pain	Body pain mannequin for previous month's pain (current). fibromyalgia mannequin (WPI) and SSSs past week's pain.
3. Foot and ankle pain	Pain present in the feet and/or ankles in the past month (current) and/or past 3 months (chronic), age at pain onset, diagnosis of foot OA, region specific *significant* foot pain (ankle, mid-foot, big toe, other toes) and age range at onset of pain. Pain Detect Questionnaire and Pain Catastrophizing Scale.
4. Injuries and operations to feet/ankles	Significant football-related injury to ankles/feet/toes. Region specific (ankle, mid-foot, big toe, other toes) total number of significant football injuries throughout career, Type of Injury (e.g. ligament rupture) and age. Significant NON-football related injury ever (same questions as above).<br><br>Injections into ankles, type of injection (cortisone, anaesthetic, don't know, or other), greatest number of injections into each ankle in a season, and throughout career. Injections elsewhere in foot, where and how many. Surgical interventions to ankle or toes, type of procedure (e.g. fracture fixation), and age. If flat-footed as informed by health professional.
5. Hallux valgus	Current and constitutional toe alignment using line drawings (straight or bunion/degree of angulation).
6. How you feel	Hospital Anxiety and Depression Scale (HADS).
7. Quality of life	Short Form-36 (SF-36) Health Survey.
8. Heading during professional football	Heading frequency during a professional match and training session. General *heading play* (minor, moderate, major) during a match. Specific 'Only Heading' training sessions

For peer review only

	ever, frequency pre-season and during season. Type of football played with during career.
9. Concussion and head injuries	Concussion Definition. Concussion diagnosis from professional football play, number of episodes, symptoms. Concussion diagnosis from other/accident/fall, number, symptoms. Serious head injury, number, diagnosis. Frequency of Head impact NOT from heading not concussive. Concussion symptoms ever from professional play (unreported), frequency, symptoms. Concussion symptoms ever (unreported) outside of professional football e.g., fall, accident, number, symptoms.
10. Memory and thinking	Current Memory problems, worsening, causing difficulty with daily function, work or socially. The Test Your memory (TYM) Questionnaire.

HBP - High Blood Pressure; WPI - Widespread Pain Index; SSS - Symptom Severity Scale; OA - osteoarthritis Pain Detect Questionnaire, Pain Catastrophizing Scale, Hospital Anxiety and Depression Scale, SF-36 Health Survey and Test Your Memory Questionnaire are referenced in the main text.

The questionnaire requests information about the individual, their medical history, smoking and alcohol usage, and all current medications whether prescribed or over the counter. A body pain mannequin is used to record pain experienced for most days of the past month anywhere in the body, and the widespread pain index (WPI) and system severity scale (SSS)[26] are included to identify symptoms and likely diagnosis of fibromyalgia. Line drawings illustrating different regions of the foot (i.e. toes, mid-foot, and heel) allow participants to specify the site of any significant pain for most days in the past month and/or over a three month period. The Pain Detect Questionnaire (PDQ), modified to focus on ankle/foot pain only, will be used to screen for neuropathic type pain,[27] and the participants' thoughts and feelings during their foot pain experience will be measured with the Pain Catastrophizing Scale.[28]

The footballers are asked to report any football-related significant injury ever sustained to their feet, ankles, or toes as one which *caused pain for most days for at least a 3-month period* and the type of injury. A similar free text and tick-box table is included which asks for any significant non-football related injuries to the feet/ankles/toes. Information regarding injections into the ankles over the course of the players career are requested via a tick box table (type of injectable, location, side) and total number ever received in any season. A similar table with a free text and tick-box asks the footballers to report any surgery to their ankle and/or toes. Control participants are asked the same set of questions about significant injury, injections, and surgery to the ankles and/or toes but without mention of football. Current toe alignment and constitutional toe alignment (in their 20's) will be assessed in all participants using validated line drawings.[29]

Anxiety and depression symptoms will be assessed using the well validated Hospital and Anxiety Depression Scale (HADS).[30 31] Quality of Life (QoL) will be measured with the well-validated RAND 36-item Health Survey 1.0 (SF-36) adapted for the physical and mental health components respectively.[32]

For peer review only

Information related to heading the ball during footballers' professional careers will be captured with detailed questions developed with our retired footballer group (see Patient and Public Involvement). This will be followed with questions related to concussion diagnoses, symptomatology, and head injuries/impacts both during professional play and outside of professional football.[34] Control participants will be asked the same questions relating to head injury and concussions but without mention of football.

Final sections will capture information on memory/cognition and include the Test Your Memory (TYM) assessment,[35] which can be self-administered for use in postal surveys (personal communication JM Brown 2019). TYM is a short cognitive test for detecting Alzheimer's disease (AD) and other cognitive problems and is reported to be more sensitive for detecting mild AD than the Mini Mental State Examination (MMSE).[35]

Telephone cognitive assessments

Telephone assessments will follow a standardised script starting with the researchers introducing themselves and the study, checking that the participant is in a room without distractions, whether they use a hearing aid, confirming consent, their name and date of birth. An initial call will be made to book in the telephone assessment at a time convenient to each participant. The validated tools described below will be administered as per published procedures.

Originally developed as a dementia screen, the Telephone Interview for Cognitive Status-modified (TICS-m) is a test for cognitive function which can be administered over the telephone.[36 37] It includes four domains on (1) orientation; (2) registration (free recall), recent memory and delayed recall (memory); (3) attention/calculation; (4) comprehension, semantic memory and repetition (language). The 13-items (maximum score 39) include: (1) day, date, season, age, telephone number; (2) a 10-word list learning exercise then free recall of that word list; (3) subtractions and counting backwards; (4) responsive naming, current reigning monarch and prime minister; (5) word opposites; (6) repetition; and (7) delayed recall of 10-word list. TICS-m is well validated for detecting a range of mild to moderate cognitive disorders with comparable sensitivity and specificity as a screening tool for dementia and Alzheimer's.[38-40]

The Verbal Fluency Test (VFT) is a short task of verbal functioning and typically consists of a task called category (or semantic) fluency where participants are given one minute to name as many unique words as possible within a semantic category (animals in this study). The category 'animals' is often used, however alternatives are 'fruits and vegetables', 'cities and towns' and 'items of clothing'. The participant's score is the number of unique correct words produced in 60 seconds.[41] Word generation deficits in those with dementia, including Alzheimer's, have been well documented.[42]

The Hopkins Verbal Learning Test (HVLT)[43] is a quick and easy to administer tool which will be used to screen for dementia. It is a word-learning test measuring episodic verbal memory, consists of three trials of free-recall of a 12-item (word) list followed by yes/no recognition from the participant. After the

For peer review only

third learning trial, the participant is read 24 words and asked to say "yes" for each word that appeared on the recall list (12 targets) and "no" for each word that did not (12 distractors). The total immediate recall (reflecting learning ability, is obtained by repeating the same word list 3 times and adding up all correctly recalled words over the 3 trials) is calculated. Good validity and reliability have been demonstrated for the HVLT, and it is cross culturally applicable and well tolerated by elderly people.[44]

Along with cognitive measures, we have included the assessment of functional activities as impairment in these is a critical feature in the diagnosis of dementia.[45] Lawton's Instrumental Activities Daily Living (IADL) scale is one of the most commonly used tools consisting of 8 items including 2 community items (mode of transport and shopping), 3 personal management (ability to use the telephone, handle money matters, and prepare one's own medication) and 3 household items (doing housekeeping, laundry, and preparing food). Responses to each of the eight items in the scale are coded as 0 (unable), 1 (partially able), or 2 (able) and are the responses summed. Summary scores range from 0 (low function, dependent) to 8 (high function, independent).[46]

Radiographic assessment

Informed written consent will be obtained prior to radiographs which will be performed by each respective NHS Trust's radiology staff. Radiographs will be taken bilaterally and weight-bearing for the foot and ankle separately. Foot dorsoplantar, medial oblique, and lateral views, and ankle anteroposterior views will be taken, as detailed in the Standard Operating Procedure (SOP). Examination and scoring of region-specific OA will be done of the hindfoot (ankle, subtalar), midfoot, and 1st metatarsophalangeal joint (MTPJ) using the La Trobe Foot Atlas and scorings.[47, 48] Those with a Kellgren and Lawrence score of less than 2 for any foot/ankle compartment and equivalent categories (i.e., completely normal, possible osteophyte or doubtful narrowing) will be defined as non-OA. Those who satisfy the definition of a KL score of greater than 2 (≥2) or equivalent for any compartment of either foot or ankle will be defined as OA.[49] Also, those with foot/ankle OA and concurrent foot/ankle pain will be defined as symptomatic foot/ankle OA.

Patient and public involvement

Questionnaire sections specific to professional football play were developed in partnership with ex-professional footballer groups, whilst those specific to sports-related concussion and memory/neurocognitive decline were developed in collaboration with concussion and dementia experts, (JM Brown).

Our retired player group contributed to the content, design and layout of the FOCUS Questionnaire. Through in-person meetings (SE), two retired professional players from Notts County Football Club directly advised (having had feedback from other retired players) specifically on the questions pertaining to:

- Heading frequency and bandings

For peer review only

- Training sessions, frequency and types
- Football types
- Types of injuries and question structure
- Head impacts and question content
- Questions on memory/cognitive function and the TYM test

The final questionnaire design was reviewed and trialled by 10 retired players at the PFA and 5 from Notts County Football Club.FC group. The time range for the ex-players to complete the questionnaire was 30-60 min and the feedback received was positive.

Outcome measures and statistical analysis

Continuous data will be presented as mean and standard deviations (SD) and categorical variables as frequencies and percentages. The statistical tests used to determine whether there is a significant difference between responses received from footballers and controls will be: t-test for normal distributions, Mann-Whitney U test for non-normal distributions, and Chi-square test for proportions with exact p values reported. Statistical significance will be set at $p<0.05$ with no allowances made for the number of statistical tests undertaken.

The prevalence of foot/ankle OA or neurodegenerative disease (and cognitive impairment) (self-reported or clinically assessed) with 95% confidence interval (CI) will be estimated per population. A Poisson regression model will be used to calculate prevalence risk ratio, adjusted for confounding factors such as age, body mass index (BMI), education, and socioecoomic status (using postcode as a proxy measure to determine the Index of Multiple Deprivation). Linear (for continuous outcome) and logistic (for dichotomous outcome) regression analyses will be used to determine risk factors related to the outcome of interests within footballers. The results of this study will follow the STROBE guidelines for the reporting of observational studies[50].

Sample size calculation

We used a value of 14.3% prevalence for foot/ankle OA in the general population[51] and an odds ratio of 2 (to be clinically meaningful) between retired footballers and general population. The sample size was calculated using the z test and a multiple logistic regression model with a correlation factor R^2 of 0.3 among multiple risk factors/covariates using G*Power V.3.1.9.2.[52] Assuming footballers would have a greater risk of foot/ankle OA than controls (one tail), with power at 90% and significance at 0.05, 210 participants per group is required for foot/ankle OA outcome. We also calculated the sample size for neurodegenerative disease using the same method. According to 6.4% prevalence for neurodegenerative diseases in the general population[53], and 3.5 times more risk in the footballers reported recently in the FIELD study[19], the sample size required is 113 per group for this outcome with power at 90% and significance at 0.05. We will therefore use the larger sample size 210 per group for

For peer review only

this study. We will recruit at least 250 retired professional footballers and 250 general population control men with a 16% leeway to account for potential dropouts and attrition.

For the postal questionnaire, we will contact all available retired professional footballers (n=900) and an age-matched sample of general population men (n=1100). Considering a 60% response rate based on our previous follow-up study[54], we would expect 540 footballers and 720 control men to respond to the questionnaire. This will provide sufficient samples from both groups to recruit the sub-sample of at least 250 participants per group required for the clinical assessments to achieve 90% power.

Discussion

To the best of our knowledge, this is the largest comparative study aiming to examine the prevalence and associated risk factors of the two common conditions, foot/ankle osteoarthritis (OA) and neurodegenerative disease in retired professional footballers versus age and sex matched general population controls. The study is expected to confirm the recent findings from the FIELD study concerning the increased risk of neurodegenerative disease in professional football players, but will also provide new evidence on whether concussion, head injury, ball type, number of games, training, position of play, are related to the increased risk of having this condition in later life. Unlike the FIELD study, this study will recruit the participants who are still alive to measure both severe disease phenotypes such as physician diagnosed dementia or Parkinson's as well as milder phenotypes such as mild cognitive impairment. Foot/ankle pain and OA will also be measured and compared between the two populations, which will further characterise the distribution of osteoarthritis and provide further evidence on the burden of foot/ankle OA in professional footballers over the general population controls following our previous study on knee OA[22]. An advantage of this 2 in 1 study is that it may help to mitigate the selection bias, which often occurs in a single disease study where people with the study condition may be more likely (or less likely) to participate. Comorbidities/multimorbidity such as cardiovascular diseases, diabetes, depression, chronic widespread pain, fibromyalgia and quality of life will also be collected to compare the general health status between footballers and matched controls.

However, the study has several limitations. Firstly, it is a cross-sectional study which can only confirm association but not causality. We have therefore planned to follow up the two cohorts for incidence of the diseases in the future. This has been considered for knee OA and multimorbidity following our previous study in knee OA, and we will be able to follow up the incidence of neurodegenerative disease/cognitive impairment and foot/ankle pain and OA as well in the future. Secondly, the study is not free from selection bias, e.g., footballers will not be selected randomly from all clubs but from our previous dataset. The same is true for the control population from the East Midlands because of the convenience of assessment in our local hospital. In addition, we are unable to randomly select participants who would like to have radiographs but need to take account of the distance to travel to the assigned NHS radiography departments. Thirdly, individuals who have developed marked cognitive and motor impairment may be unable or decline to respond to the questionnaire, leading to selection bias

For peer review only

towards milder conditions. Last but not least, the COVID-19 pandemic has affected the project and protocols. We were unable to *conduct* face-to-face assessments of cognitive and motor function, or to obtain radiographs (Nottingham, Southampton, Salford, Leeds, and Imperial College London). The project was temporarily halted following the national lockdown due to the COVID pandemic in March 2020. Following management committee meetings and advice from the University of Nottingham governance/Research Ethics Committee/Health Research Authority teams, it was decided to amend the original protocol from in-person assessments to telephone assessments in order to enable the study to begin. This approach was not possible for some of the neurocognitive assessments, and these were replaced with the TICS-m.[36 37] The TICS-m uses questions from MOCA and MMSE, with which it is highly correlated; it is a much shorter assessment and can be administered by telephone so is safer for all involved during the pandemic and lockdown. The VFT, HVLT, and IADL[41 43 45 46] were also retained as they can be administered by telephone, however the planned clinical neurological assessments (motor function) were removed.

Although the gold standard for testing cognitive function, and diagnosis of mild cognitive impairment (MCI) and dementia is face-to-face assessment using a battery of standardised and validated cognitive tests,[55] these are not without their drawbacks.[56] TICS-m is the most frequently used telephone-based cognitive screening test in medium-large studies and epidemiological surveys.[55] Key advantages include little evidence of ceiling and/or practice effects, and its better acceptance by participants who find the telephone interview less threatening than a face-to-face battery of cognitive tests in clinic.[36] Although hearing and communication may pose an issue for some, this can be mitigated by a skilled interviewer.[36 57] In contrast, the foot/ankle radiographs require NHS clinical radiology services, these have to be fully suspended until research activities within Hospital trusts are resumed.

In conclusion, we expect that the results of our study will establish prevalence of foot and ankle OA symptoms; radiographic prevalence of foot and ankle OA; and prevalence of cognitive impairment and neurodegenerative diseases in ex-professional footballers compared to general population controls. Thus, providing the much-needed answers following on from recent evidence that professional footballers had a 3.5 times higher death rate from neurodegenerative diseases compared with population controls.[19] Given that football is played professionally and recreationally in over 200 countries by more than 250 million people,[58] robust evidence on mental and physical health and well-being in retired professional footballers is a vital first step in establishing risk profiles, which will assist in developing effective prevention strategies for mitigating against longer-term health consequences.

Ethics and dissemination

All study aspects were approved by the East Midlands-Leicester Central Research Ethics Committee and HRA (REC ref: 19/EM/0354) on 23 January 2020. Study results will be submitted to the FA, Versus Arthritis, regulatory authorities, and peer reviewed journals for publication and presented at national and international conferences. Study participants will be provided with any resulting publications at their request.

For peer review only - http://bmjopen.bmj.com/site/about/guidelines.xhtml

Data statement

All data will be collected and stored in accordance with the Data Protection Act, 2018, University of Nottingham and NHS data handling and maintenance guidelines. The CRF will only collect the minimum required information for the purposes of the study. The study will be conducted in accordance with the ethical principles that have their origin in the Declaration of Helsinki, 1996; the principles of Good Clinical Practice and the UK Department of Health Policy Framework for Health and Social Care, 2017.

Authors' contributions

SE drafted, revised, finalised the manuscript, implemented (conducted) the study and NHS Trust collaborating centres. SE and GSF designed and developed the Footballer Questionnaires. GSF, MD, WZ conceptualised the study. All authors (SE, GSF, EH, AT, MB, CWF, GF, EF, TB, MD, WZ) were involved in the design and development of the study protocols. SE, AT, EH and WZ collected data. All authors reviewed the draft and approved the final manuscript. WZ is the guarantor for the project.

Acknowledgments

We are grateful to Mr Les Bradd and all other retired professional footballers who gave their time and expertise to contribute to this study, including those from Notts County Football Club, The PFA, especially Mr Richard Jobson, and all our PPI group. We also extend our gratitude to Dr Jerry M Brown for his helpful discussion and invaluable advice on the TYM test, and our collaborators Professors Cathy Bowen, and Terry O'Neill, and Drs Richard Wakefield, Mo Aslam, and Lucy Gates. We are indebted to Dr Charlotte Cowie for her guidance and support throughout the past few years. We wish to acknowledge the FA, the PFA, Versus Arthritis, and the University of Nottingham for supporting this study, and King Abdulaziz University, Jeddah, Saudi Arabia for sponsoring AT's PhD programme. Finally, we are thankful to our participants both from the retired professional football players and those from the general population dwelling adults from the East-Midlands. Without their pertinent and persistent support over the past years, we would not be able to undertake this study especially during the COVID-19 pandemic/lockdown.

Figure Legend

Figure 1: Flow chart of FOCUS study stages and procedures. *Minimum sample size according to sample size calculation.

Funding statement

This work is supported by grants from Versus Arthritis, grant number (21595) and The Football Association (FA), grant number (not applicable).

For peer review only

Competing interests.

CF provides consultancy services to the English Premier League.

Patient consent for publication. Not required

Provenance and peer review. Not commissioned, externally peer reviewed for ethical and funding approval prior to submission.

References

1. Drawer S, Fuller CW. Propensity for osteoarthritis and lower limb joint pain in retired professional soccer players. *Br J Sports Med* 2001;35(6):402-8. doi: 10.1136/bjsm.35.6.402 [published Online First: 2001/12/01]
2. Hawkins RD, Fuller CW. A prospective epidemiological study of injuries in four English professional football clubs. *British journal of sports medicine* 1999;33(3):196-203. doi: 10.1136/bjsm.33.3.196
3. Ekstrand J, Hägglund M, Waldén M. Injury incidence and injury patterns in professional football: the UEFA injury study. *Br J Sports Med* 2011;45(7):553-8. doi: 10.1136/bjsm.2009.060582 [published Online First: 2009/06/26]
4. López-Valenciano A, Ruiz-Pérez I, Garcia-Gómez A, et al. Epidemiology of injuries in professional football: a systematic review and meta-analysis. *British journal of sports medicine* 2020;54(12):711. doi: 10.1136/bjsports-2018-099577
5. Chomiak J, Junge A, Peterson L, et al. Severe injuries in football players. Influencing factors. *The American journal of sports medicine* 2000;28(5 Suppl):S58-68. doi: 10.1177/28.suppl_5.s-58 [published Online First: 2000/10/14]
6. Waldén M, Hägglund M, Ekstrand J. Time-trends and circumstances surrounding ankle injuries in men's professional football: an 11-year follow-up of the UEFA Champions League injury study. *Br J Sports Med* 2013;47(12):748-53. doi: 10.1136/bjsports-2013-092223 [published Online First: 2013/07/03]
7. Jain N, Murray D, Kemp S, et al. Frequency and trends in foot and ankle injuries within an English Premier League Football Club using a new impact factor of injury to identify a focus for injury prevention. *Foot Ankle Surg* 2014;20(4):237-40. doi: 10.1016/j.fas.2014.05.004 [published Online First: 2014/12/03]
8. Kuijt MT, Inklaar H, Gouttebarge V, et al. Knee and ankle osteoarthritis in former elite soccer players: a systematic review of the recent literature. *J Sci Med Sport* 2012;15(6):480-7. doi: 10.1016/j.jsams.2012.02.008 [published Online First: 2012/05/11]
9. Gouttebarge V, Aoki H, Kerkhoffs G. Lower extremity osteoarthritis is associated with lower health-related quality of life among retired professional footballers. *Phys Sportsmed* 2018;46(4):471-76. doi: 10.1080/00913847.2018.1451718 [published Online First: 2018/03/13]
10. Turner A, Barlow J, Ilbery B. Play Hurt, Live Hurt: Living with and Managing Osteoarthritis from the Perspective of Ex-professional Footballers. *J Health Psychol* 2002;7(3):285-301. doi: 10.1177/1359105302007003222 [published Online First: 2002/05/01]
11. Turner AP, Barlow JH, Heathcote-Elliott C. Long term health impact of playing professional football in the United Kingdom. *British journal of sports medicine* 2000;34(5):332-36. doi: 10.1136/bjsm.34.5.332
12. Fernandes GS, Parekh SM, Moses J, et al. Depressive symptoms and the general health of retired professional footballers compared with the general population in the UK: a case-control study. *BMJ Open* 2019;9(9):e030056. doi: 10.1136/bmjopen-2019-030056 [published Online First: 2019/09/11]
13. Rutherford A, Stewart W, Bruno D. Heading for trouble: is dementia a game changer for football? *British journal of sports medicine* 2019;53(6):321-22. doi: 10.1136/bjsports-2017-097627
14. Guskiewicz KM, Marshall SW, Bailes J, et al. Association between recurrent concussion and late-life cognitive impairment in retired professional football players. *Neurosurgery*

For peer review only

2005;57(4):719-26; discussion 19-26. doi: 10.1093/neurosurgery/57.4.719 [published Online First: 2005/10/22]

15. Lee MJ. Increase risk for neurodegenerative diseases in professional athletes. University of Boston, 2015.
16. Ling H, Morris HR, Neal JW, et al. Mixed pathologies including chronic traumatic encephalopathy account for dementia in retired association football (soccer) players. *Acta Neuropathol* 2017;133(3):337-52. doi: 10.1007/s00401-017-1680-3 [published Online First: 2017/02/17]
17. Fann JR, Ribe AR, Pedersen HS, et al. Long-term risk of dementia among people with traumatic brain injury in Denmark: a population-based observational cohort study. *Lancet Psychiatry* 2018;5(5):424-31. doi: 10.1016/s2215-0366(18)30065-8 [published Online First: 2018/04/15]
18. Fuller CW, Junge A, Dvorak J. A six year prospective study of the incidence and causes of head and neck injuries in international football. *Br J Sports Med* 2005;39 Suppl 1(Suppl 1):i3-9. doi: 10.1136/bjsm.2005.018937 [published Online First: 2005/07/28]
19. Mackay DF, Russell ER, Stewart K, et al. Neurodegenerative Disease Mortality among Former Professional Soccer Players. *New England Journal of Medicine* 2019;381(19):1801-08. doi: 10.1056/NEJMoa1908483
20. Kontos AP, Braithwaite R, Chrisman SPD, et al. Systematic review and meta-analysis of the effects of football heading. *Br J Sports Med* 2017;51(15):1118-24. doi: 10.1136/bjsports-2016-096276 [published Online First: 2016/12/23]
21. Tarnutzer AA, Straumann D, Brugger P, et al. Persistent effects of playing football and associated (subconcussive) head trauma on brain structure and function: a systematic review of the literature. *Br J Sports Med* 2017;51(22):1592-604. doi: 10.1136/bjsports-2016-096593 [published Online First: 2016/11/07]
22. Fernandes GS, Parekh SM, Moses J, et al. Prevalence of knee pain, radiographic osteoarthritis and arthroplasty in retired professional footballers compared with men in the general population: a cross-sectional study. *Br J Sports Med* 2018;52(10):678-83. doi: 10.1136/bjsports-2017-097503 [published Online First: 2017/11/05]
23. Parekh SM, Fernandes GS, Moses JP, et al. Risk Factors for Knee Osteoarthritis in Retired Professional Footballers: A Cross-Sectional Study. *Clinical journal of sport medicine : official journal of the Canadian Academy of Sport Medicine* 2019 doi: 10.1097/JSM.0000000000000742 [published Online First: 2019/06/04]
24. Faul F, Erdfelder E, Lang A-G, et al. G*Power 3: A flexible statistical power analysis program for the social, behavioral, and biomedical sciences. *Behavior Research Methods* 2007;39(2):175-91. doi: 10.3758/BF03193146
25. Fernandes GS, Sarmanova A, Warner S, et al. Knee pain and related health in the community study (KPIC): a cohort study protocol. *BMC Musculoskelet Disord* 2017;18(1):404. doi: 10.1186/s12891-017-1761-4
26. Wolfe F, Clauw DJ, Fitzcharles MA, et al. Fibromyalgia criteria and severity scales for clinical and epidemiological studies: a modification of the ACR Preliminary Diagnostic Criteria for Fibromyalgia. *J Rheumatol* 2011;38(6):1113-22. doi: 10.3899/jrheum.100594 [published Online First: 2011/02/03]
27. Freynhagen R, Baron R, Gockel U, et al. painDETECT: a new screening questionnaire to identify neuropathic components in patients with back pain. *Curr Med Res Opin* 2006;22(10):1911-20. doi: 10.1185/030079906x132488 [published Online First: 2006/10/07]
28. Sullivan MJL, Bishop SR, Pivik J. The Pain Catastrophizing Scale: Development and validation. *Psychological Assessment* 1995;7(4):524-32. doi: 10.1037/1040-3590.7.4.524
29. Chatterton BD, Muller S, Thomas MJ, et al. Inter and intra-rater repeatability of the scoring of foot pain drawings. *Journal of Foot and Ankle Research* 2013;6(1):44. doi: 10.1186/1757-1146-6-44
30. Spinhoven P, Ormel J, Sloekers PP, et al. A validation study of the Hospital Anxiety and Depression Scale (HADS) in different groups of Dutch subjects. *Psychol Med* 1997;27(2):363-70. doi: 10.1017/s0033291796004382 [published Online First: 1997/03/01]
31. Bjelland I, Dahl AA, Haug TT, et al. The validity of the Hospital Anxiety and Depression Scale. An updated literature review. *J Psychosom Res* 2002;52(2):69-77. doi: 10.1016/s0022-3999(01)00296-3 [published Online First: 2002/02/08]
32. Ware JE, Jr., Sherbourne CD. The MOS 36-item short-form health survey (SF-36). I. Conceptual framework and item selection. *Med Care* 1992;30(6):473-83. [published Online First: 1992/06/11]

For peer review only

33. Medical Outcomes Study: 36-item Short Form Survey [Available from: https://www.rand.org/health-care/surveys_tools/mos/36-item-short-form.html.
34. McCrory P, Meeuwisse W, Dvorak J, et al. Consensus statement on concussion in sport—the 5th international conference on concussion in sport held in Berlin, October 2016. *British journal of sports medicine* 2017;51(11):838-47. doi: 10.1136/bjsports-2017-097699
35. Brown J, Pengas G, Dawson K, et al. Self administered cognitive screening test (TYM) for detection of Alzheimer's disease: cross sectional study. *Bmj* 2009;338:b2030. doi: 10.1136/bmj.b2030 [published Online First: 2009/06/11]
36. Brandt J, Spencer M, Folstein M. The Telephone Interview for Cognitive Status. *Neuropsychiatry, Neuropsychology, & Behavioral Neurology* 1988;1(2):111-17.
37. de Jager CA, Budge MM, Clarke R. Utility of TICS-M for the assessment of cognitive function in older adults. *Int J Geriatr Psychiatry* 2003;18(4):318-24. doi: 10.1002/gps.830 [published Online First: 2003/04/04]
38. Smith MM, Tremont G, Ott BR. A review of telephone-administered screening tests for dementia diagnosis. *Am J Alzheimers Dis Other Demen* 2009;24(1):58-69. doi: 10.1177/1533317508327586 [published Online First: 2009/01/01]
39. Welsh KA, Breitner JC, Magruder-Habib KM. Detection of dementia in the elderly using telephone screening of cognitive status. *Neuropsychiatry, Neuropsychology, & Behavioral Neurology* 1993;6(2):103-10.
40. Wolfson C, Kirkland SA, Raina PS, et al. Telephone-administered cognitive tests as tools for the identification of eligible study participants for population-based research in aging. *Can J Aging* 2009;28(3):251-9. doi: 10.1017/s0714980809990092 [published Online First: 2009/10/29]
41. Alegret M, Peretó M, Pérez A, et al. The Role of Verb Fluency in the Detection of Early Cognitive Impairment in Alzheimer's Disease. *J Alzheimers Dis* 2018;62(2):611-19. doi: 10.3233/jad-170826 [published Online First: 2018/02/27]
42. Gomez RG, White DA. Using verbal fluency to detect very mild dementia of the Alzheimer type. *Arch Clin Neuropsychol* 2006;21(8):771-5. doi: 10.1016/j.acn.2006.06.012 [published Online First: 2006/10/03]
43. Brandt J. The hopkins verbal learning test: Development of a new memory test with six equivalent forms. *Clinical Neuropsychologist* 1991;5(2):125-42. doi: 10.1080/13854049108403297
44. Hogervorst E, Combrinck M, Lapuerta P, et al. The Hopkins Verbal Learning Test and screening for dementia. *Dement Geriatr Cogn Disord* 2002;13(1):13-20. doi: 10.1159/000048628 [published Online First: 2001/12/04]
45. Association AP. Diagnostic and Statistical Manual of Metal Disorders. Fifth Edition ed. Arlington VA: American Psychiatric Publishing 2013.
46. Lawton MP, Brody EM. Assessment of Older People: Self-Maintaining and Instrumental Activities of Daily Living1. *The Gerontologist* 1969;9(3_Part_1):179-86. doi: 10.1093/geront/9.3_Part_1.179
47. Menz HB, Munteanu SE, Landorf KB, et al. Radiographic classification of osteoarthritis in commonly affected joints of the foot. *Osteoarthritis Cartilage* 2007;15(11):1333-8. doi: 10.1016/j.joca.2007.05.007 [published Online First: 2007/07/13]
48. Kraus VB, Kilfoil TM, Hash TW, 2nd, et al. Atlas of radiographic features of osteoarthritis of the ankle and hindfoot. *Osteoarthritis Cartilage* 2015;23(12):2059-85. doi: 10.1016/j.joca.2015.08.008 [published Online First: 2015/09/01]
49. Kellgren JH, Lawrence JS. Radiological assessment of osteo-arthrosis. *Ann Rheum Dis* 1957;16(4):494-502. doi: 10.1136/ard.16.4.494 [published Online First: 1957/12/01]
50. von Elm E, Altman DG, Egger M, et al. The Strengthening the Reporting of Observational Studies in Epidemiology (STROBE) statement: guidelines for reporting observational studies. *The Lancet* 2007;370(9596):1453-57. doi: 10.1016/S0140-6736(07)61602-X
51. Roddy E, Thomas MJ, Marshall M, et al. The population prevalence of symptomatic radiographic foot osteoarthritis in community-dwelling older adults: cross-sectional findings from the Clinical Assessment Study of the Foot. *Annals of the Rheumatic Diseases* 2015;74(1):156. doi: 10.1136/annrheumdis-2013-203804
52. Faul F, Erdfelder E, Buchner A, et al. Statistical power analyses using G*Power 3.1: Tests for correlation and regression analyses. *Behavior Research Methods* 2009;41(4):1149-60. doi: 10.3758/BRM.41.4.1149
53. Lobo A, Launer LJ, Fratiglioni L, et al. Prevalence of dementia and major subtypes in Europe: A collaborative study of population-based cohorts. Neurologic Diseases in the Elderly Research Group. *Neurology* 2000;54(11 Suppl 5):S4-9. [published Online First: 2000/06/15]

For peer review only - http://bmjopen.bmj.com/site/about/guidelines.xhtml

54. Ingham SL, Zhang W, Doherty SA, et al. Incident knee pain in the Nottingham community: a 12-year retrospective cohort study. *Osteoarthritis Cartilage* 2011;19(7):847-52. doi: 10.1016/j.joca.2011.03.012
55. Herr M, Ankri J. A critical review of the use of telephone tests to identify cognitive impairment in epidemiology and clinical research. *J Telemed Telecare* 2013;19(1):45-54. doi: 10.1177/1357633x12474962 [published Online First: 2013/02/08]
56. Pachana NA, Alpass FM, Blakey JA, et al. A comparison of the MMSE and the TICS-m in hearing-impaired older adults. *Australasian Journal on Ageing* 2006;25(2):89-93. doi: https://doi.org/10.1111/j.1741-6612.2006.00156.x
57. Kliegel M, Martin M, Jäger T. Development and validation of the Cognitive Telephone Screening Instrument (COGTEL) for the assessment of cognitive function across adulthood. *J Psychol* 2007;141(2):147-70. doi: 10.3200/jrlp.141.2.147-172 [published Online First: 2007/05/08]
58. Kunz M. 265 million playing football. FIFA Magazine. Zurich: FIFA 2007 [Available from: https://condorperformance.com/wp-content/uploads/2020/02/emaga_9384_10704.pdf.

For peer review only

Figure 1 Flow chart of FOCUS study stages and procedures. * Minimum sample size according to the sample size calculation

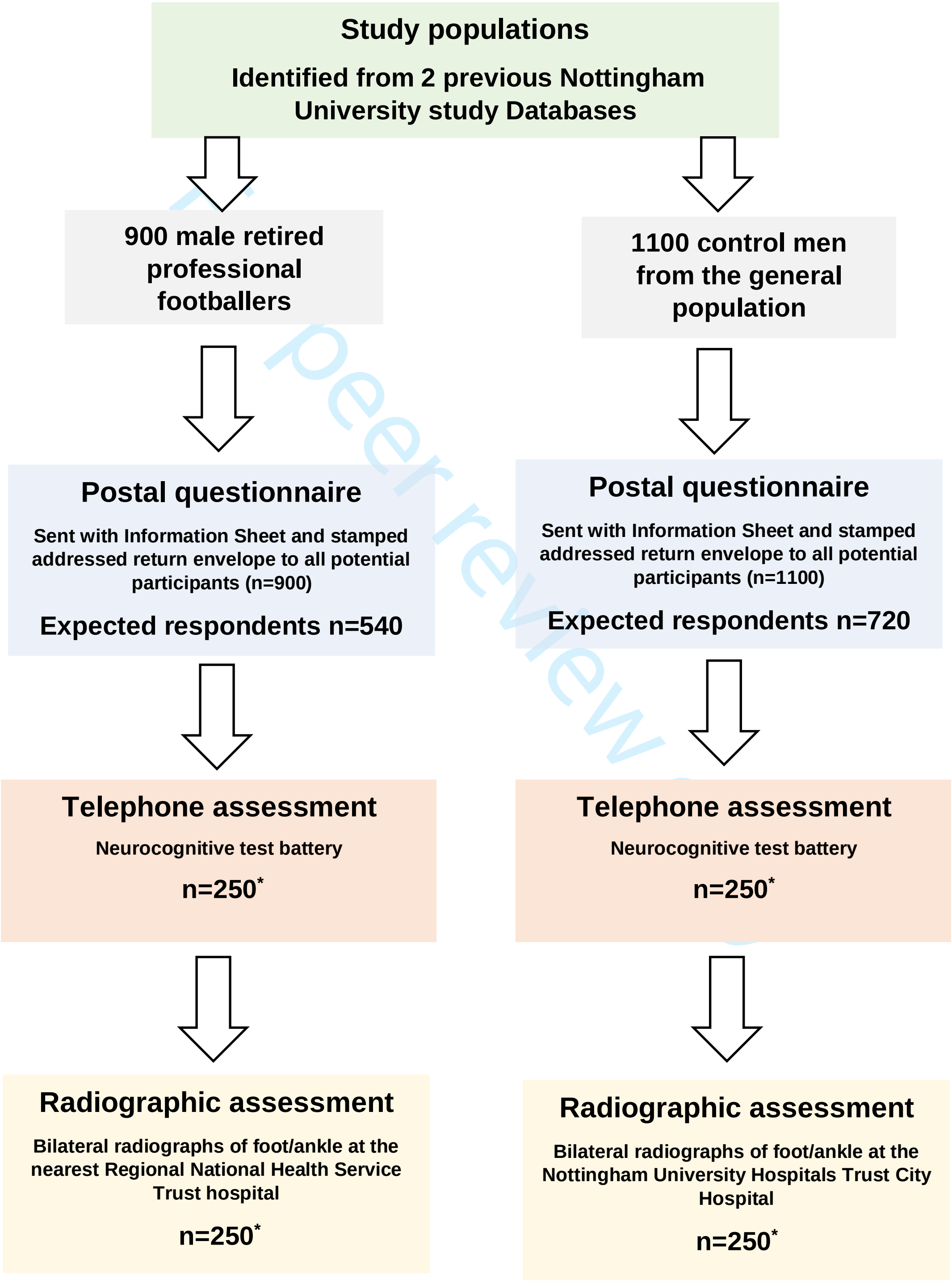

Supplement: Author's manuscript [file bmjopen-2021-054371.draft_revisions.pdf]
